# Genomic conservation of crop wild relatives: A case study of citrus

**DOI:** 10.1371/journal.pgen.1010811

**Published:** 2023-06-20

**Authors:** Nan Wang, Shuo Cao, Zhongjie Liu, Hua Xiao, Jianbing Hu, Xiaodong Xu, Peng Chen, Zhiyao Ma, Junli Ye, Lijun Chai, Wenwu Guo, Robert M. Larkin, Qiang Xu, Peter L. Morrell, Yongfeng Zhou, Xiuxin Deng

**Affiliations:** 1 National Key Laboratory for Germplasm Innovation & Utilization of Horticultural Crops, Huazhong Agricultural University, Wuhan, China; 2 State Key Laboratory of Tropical Crop Breeding, Shenzhen Branch, Guangdong Laboratory of Lingnan Modern Agriculture, Key Laboratory of Synthetic Biology, Ministry of Agriculture and Rural Affairs, Agricultural Genomics Institute at Shenzhen, Chinese Academy of Agricultural Sciences, Shenzhen, China; 3 Hubei Hongshan Laboratory, Wuhan, China; 4 Institute of Horticultural Research, Hunan Academy of Agricultural Sciences, Changsha, China; 5 Department of Agronomy and Plant Genetics, University of Minnesota, St. Paul, Minnesota, United States of America; 6 State Key Laboratory of Tropical Crop Breeding, Tropical Crops Genetic Resources Institute, Chinese Academy of Tropical Agricultural Sciences, Haikou, China; Peking University, CHINA

## Abstract

Conservation of crop wild relatives is critical for plant breeding and food security. The lack of clarity on the genetic factors that lead to endangered status or extinction create difficulties when attempting to develop concrete recommendations for conserving a citrus wild relative: the wild relatives of crops. Here, we evaluate the conservation of wild kumquat (*Fortunella hindsii*) using genomic, geographical, environmental, and phenotypic data, and forward simulations. Genome resequencing data from 73 accessions from the *Fortunella* genus were combined to investigate population structure, demography, inbreeding, introgression, and genetic load. Population structure was correlated with reproductive type (i.e., sexual and apomictic) and with a significant differentiation within the sexually reproducing population. The effective population size for one of the sexually reproducing subpopulations has recently declined to ~1,000, resulting in high levels of inbreeding. In particular, we found that 58% of the ecological niche overlapped between wild and cultivated populations and that there was extensive introgression into wild samples from cultivated populations. Interestingly, the introgression pattern and accumulation of genetic load may be influenced by the type of reproduction. In wild apomictic samples, the introgressed regions were primarily heterozygous, and genome-wide deleterious variants were hidden in the heterozygous state. In contrast, wild sexually reproducing samples carried a higher recessive deleterious burden. Furthermore, we also found that sexually reproducing samples were self-incompatible, which prevented the reduction of genetic diversity by selfing. Our population genomic analyses provide specific recommendations for distinct reproductive types and monitoring during conservation. This study highlights the genomic landscape of a wild relative of citrus and provides recommendations for the conservation of crop wild relatives.

## Author summary

Conservation genomics offers a comprehensive approach to understand the underlying genetic and environmental factors affecting the conservation of species. Despite its importance, the conservation genomics of most crop wild relatives remains poorly understood. In this study, we investigated the population fragmentation, inbreeding, gene flow, and genetic load of a citrus wild relative, *Fortunella hindsii*, using a combination of genomic, geographical, environmental, and phenotypic data, as well as forward simulations. *Fortunella hindsii*, listed on the registry of National Key Protected Wild Plants in China, has two types of reproduction, sexual and apomictic. Conservation genomics provided insights into the genetic diversity and structure, which are critical for developing effective conservation strategies. Our analysis also helped to assess the risks of hybridization and introgression from cultivated to wild populations. We found that different patterns of introgression and genetic load may be influenced by reproductive type; for example, deleterious variants may hide in the heterozygous state in apomictic populations. Sexually reproducing samples with the self-incompatibility mechanism can prevent rapid loss of genetic diversity caused by selfing. This study serves as an example of conservation genomics and the importance of utilizing important wild relatives of crops to inform broader conservation efforts.

## Introduction

The intensive farming of domesticated crops has led to the dramatic decline and fragmentation of populations of crop wild progenitors [1] and, sometimes, even to extinction [2]. More than 20 wild relatives of rice, wheat, and yam have been listed as threatened species on the latest version of the International Union for Conservation of Nature (IUCN) Red List (https://www.iucn.org/). Crop wild relatives play a crucial role in crop breeding and food security because they frequently contain beneficial traits absent in cultivars [3]. For example, flavor and metabolites were lost during the domestication of tomatoes. However, introgression experiments with a wild relative restored some of the lost metabolites [4]. Evidence for the great potential of the wild relatives of crops was also provided by the *de novo* domestication of rice from a wild allotetraploid rice [5]. The conservation of the wild relatives of crops is of critical importance for the medium to long-term security of the human food supply [6]. However, the conservation status of most of the world’s wild relatives of agricultural species remains poorly understood. Genomic approaches have been used successfully in the conservation of wild organisms, such as island foxes [7], wolves [8], and the ironwood tree *Ostrya rehderiana* [9]. Conservation genomics efforts provide at least two types of data that enhance our understanding of the specific challenges that obstruct the conservation of individual species.

First, genomic data can be used to gain insight into stochastic demographic and environmental processes and to elucidate genetic components, such as the effects of inbreeding and genetic load in a small population [10]. The decline in population size and associated changes in the effective number of breeding individuals (i.e., effective population size (*N*_e_)) leads to the reduction of genetic diversity [11] and in turn to concomitant increases in genetic drift [12]. At the same time, the transition of reproductive patterns from outcrossing to inbreeding is associated with a dramatic reduction in recombination efficiency, which reduces the efficacy of purifying selection [13]. Therefore, the accumulation of deleterious variants, known as genetic load, can reduce the viability of small and isolated populations [14]. Although the high levels of inbreeding increase homozygosity, it is possible to antagonize the purging of exposed recessive deleterious alleles [15].

Second, genomic analysis can track uncontrolled gene flow from cultivated to wild populations, a major concern for conservation [13]. Introgression is an evolutionary force that promotes genetic homogeneity among populations that might lead to genetic swamping, the replacement of wild relatives with hybrids [16–18] and evolutionary trajectories influenced by the dynamics of the gene flow, natural selection and genetic drift [19]. In this scenario, the accumulation of deleterious mutations may counteract the effects of adaptive selection and lead to outbreeding depression [20]. The *N*_*e*_ is small in an endangered population. Therefore, genetic drift is expected to increase the frequency of disadvantageous alleles as introgression continues [21]. Furthermore, hybridization and introgression are common because of the relatively short divergence times between modern crops and their wild relatives and because reproductive isolation between them is often incomplete [17,22], especially for perennial crops with long generation times [23]. Moreover, there may also be insufficient time for differentiation of the ecological niche occupied by crops and their wild relatives.

Citrus is an important perennial fruit crop. Multiple closely-related but distinct cultigens of citrus are grown in more than 140 countries around the world [24]. The ten major commercial species belong to two genera, *Citrus* and *Fortunella*. Changes in the environment led to extreme declines in *Citrus* populations. Indeed, some wild relatives now exist only as individual trees [25]. In contrast, small populations of wild *Fortunella* still exist [26]. This situation has become more apparent during the past two decades. The genus of *Fortunella* includes cultivated kumquat (*F*. *crassifolia* and *F*. *japonica*) and wild kumquat (*F*. *hindsii*, also known as Hongkong kumquat) [27]. *F*. *hindsii* is distributed across Guangdong, Fujian, Zhejiang, and Jiangxi provinces in China, south of the Nanling Mountains [28]. Phylogenetic analyses based on cytoplasmic SNPs, nuclear SSRs and whole genome sequences indicate that cultivated kumquat is closely related to wild kumquat [26,28,29]. Recently, *F*. *hindsii* was listed as endangered on the List of National Key Protected Wild Plants in China (https://www.forestry.gov.cn/). The two types of reproduction found in various citrus varieties (i.e., sexual reproduction and facultative apomixis) are found in both the wild and cultivated populations of *Fortunella* [27]. Facultative apomictic kumquat use an adventitious embryonic process to produce offspring genotypically identical to the maternal lineage and, at a low frequency, progeny from sexual reproduction, in which sexual and apomictic processes co-exist [30]. This leaky sexual reproduction is important in that it helps apomicts to resist the effects of Muller’s ratchet [31]. Although some investigations into wild kumquat have been performed, conservation initiatives have overlooked reproductive phenotypes. In this study, we used the genus *Fortunella* (commonly known as kumquat) as a genetic system to study the conservation of citrus wild relatives. We collected short reads from 73 accessions from the *Fortunella* genus to evaluate population structure, demography and inbreeding. Furthermore, these data were combined with biogeographical data to investigate the ecological niche overlap, introgression, and genetic load. We considered the different reproductive types in our genomic conservation analyses and practices of wild *Fortunella*. Our study aims to answer five questions: 1) Is there a clear divergence in the ecological niche occupied by wild and cultivated populations? 2) How much genetic diversity is present in wild populations, and how do levels of inbreeding and population demography impact genetic diversity? 3) Does genetic evidence support the ongoing gene flow between wild and cultivated populations? If gene flow has continued, what are the genetic effects at the genomic level? 4) Two different reproductive types, apomixis and sexual reproduction, coexist in wild populations. How do the different reproductive types impact the deleterious load in the endangered wild species? 5) Can insight from population genomics help provide concrete recommendations for wild citrus species conservation practices?

## Results

### Overlapping distributions and ecological niches of wild and cultivated *Fortunella*

**Fig 1:**
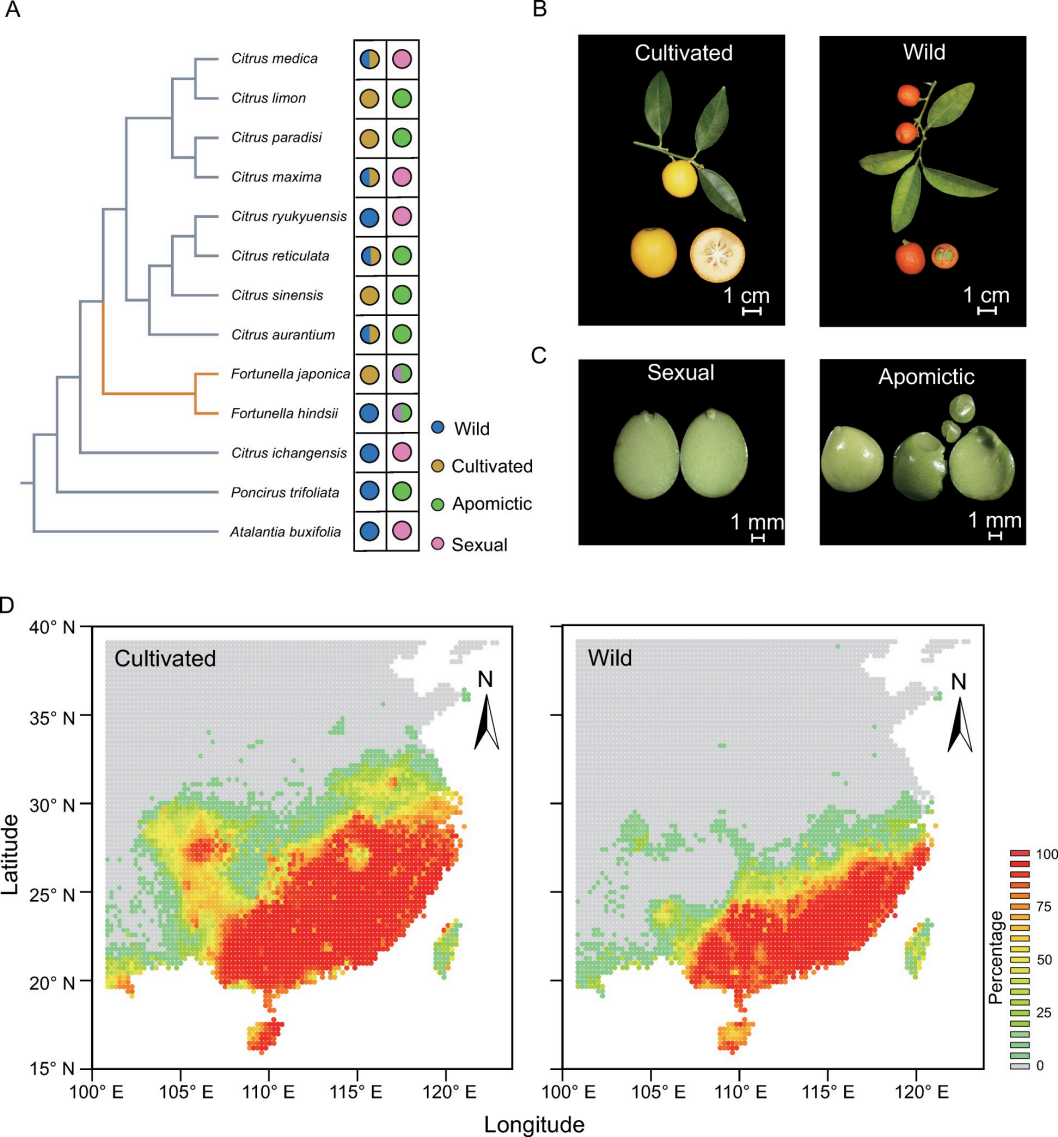
Climatic niche for wild and cultivated *Fortunella*. (A) Phylogeny of 13 citrus species including cultivars and their wild relatives. The different reproductive types are indicated with different colors. Fortunella species capable of both apomixis and sexual reproduction are highlighted. (B) Cultivated and wild kumquats. Scale bars = 1 cm. There are two reproductive types for kumquat, sexually reproducing and apomictic. Scale bars = 1 mm. (C) Predicted distribution of wild and cultivated kumquats. The probabilities of the predicted distributions are represented with a color gradient. The base layer of the map from Tianditu, the National Platform for Common Geospatial Information Services (NPCGIS) https://www.tianditu.gov.cn/. The base layer is under CC BY 4.0 license.

To elucidate a picture of *Fortunella* conservation, we constructed the phylogeny of *Citrinae* and highlighted the reproductive types and wild populations and individuals (Figs 1A, S1 and S2). The co-existence of different reproductive types—apomictic and sexual reproduction—in cultivated and wild *Fortunella* was prominent during the diversification of citrus (Fig 1B and 1C). The pertinent question for conservation is whether there is an ecological relatedness between the cultivated and wild species. Therefore, we assessed over a half-century of collective geographic data for *Fortunella* from a Chinese Virtual Herbarium. We analyzed the overlap in ecological niches based on 19 bioclimatic variables from WorldClim [32]. The 396 geographical records for 151 cultivated samples and 245 wild samples and the pertinent climate information were collected (S1 and S2 Tables and S3 Fig). Subsequently, the estimated overlapping probability (~58%) from a principal component analysis (PCA) (PC1, 47.5% and PC2, 20.6%) provided evidence for substantial overlap of distributional and climatic niches for cultivated and wild kumquats (S4 and S5 Figs). To identify the candidate abiotic factors related to the distributions of the wild and cultivated populations, we focused on the most informative environmental variables for distributional difference based on least absolute shrinkage and selection operator (LASSO) regression (S6 Fig, see Methods). Four environmental variables (temperature seasonality, mean temperature of the driest quarter, annual precipitation and precipitation during the coldest quarter) establish the niche region for wild and cultivated kumquats and that might influence flowering (i.e., flowering time from late spring to early summer [33]) and fruit development (i.e., approximately 10 to 15 cm of water per month in the summer [34]). To outline potentially habitable regions for wild and cultivated *Fortunella*, we independently predicted the distribution with two different methods (Fig 1D, see Methods). The heatmap is consistent with the Nanling Mountains (24°-26° N, 110°-115° E) serving as the northern border for the geographical range of wild kumquats [26]. The geographical range was extended for cultivated kumquats into higher latitudes than wild relatives, corresponding to the estimated overlapping probability (S7 Fig) and the prediction of MAXENT (S8 Fig). Although the isolation facilities on intensive farms limit production areas, the geographical distribution and ecological niche modeling revealed overlapping ecological distributions for cultivated and wild kumquats. Based on these data, we suggest that geographic barriers to gene flow may be absent (i.e., pollinator activity) and that the overlapping geographic range is a major concern for the conservation of wild kumquat.

### Effects of population structure and demographic history

**Fig 2:**
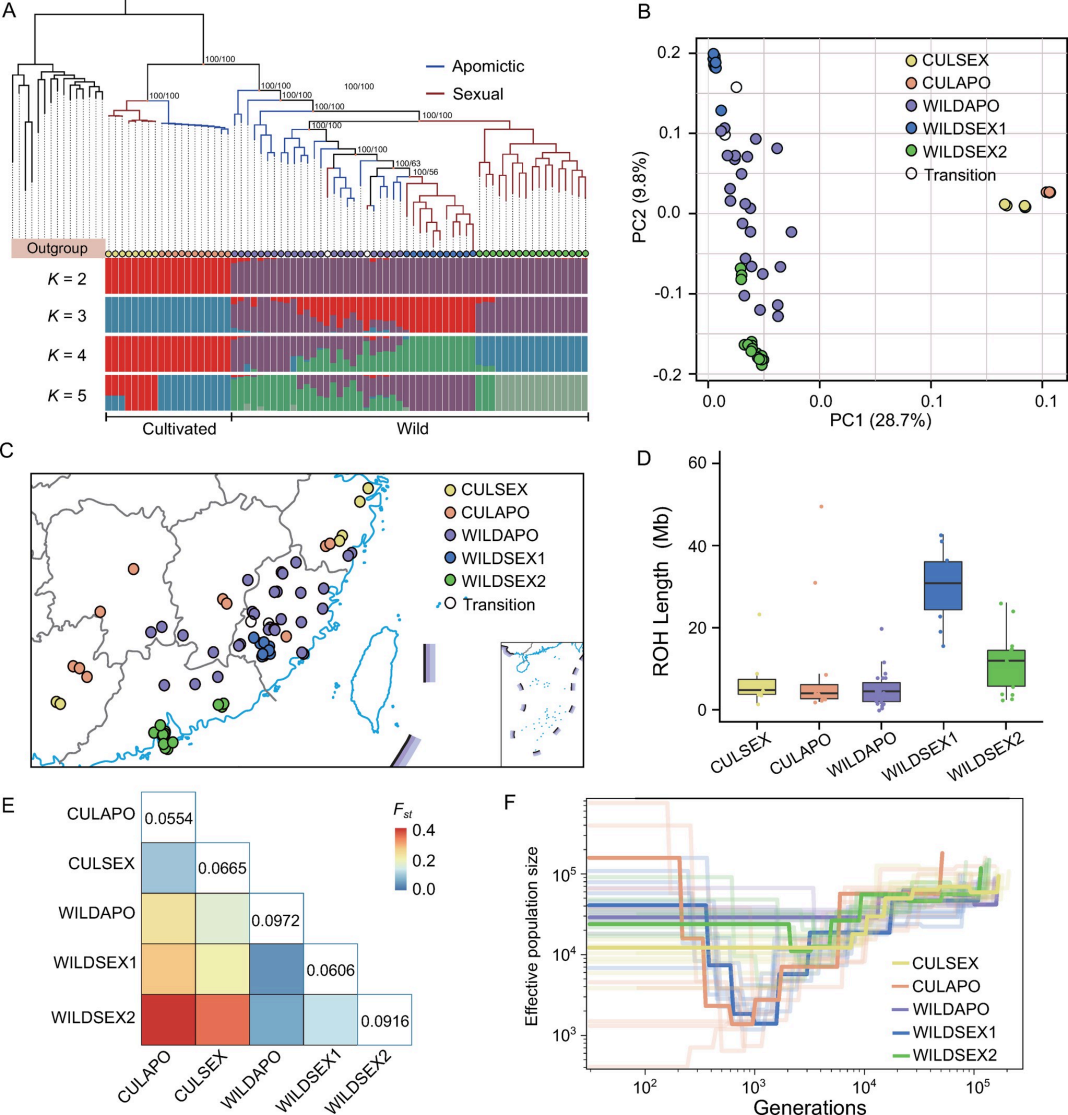
Population structure and demographic history. (a) Phylogenetic tree and population ancestry of *Fortunella*. The different reproductive types in cultivated and wild kumquats are presented with different colored branches. The estimated admixture proportions ranged from *K* = 2 to *K* = 5. (B) Principal component analysis (PCA) of 73 sequenced samples in *Fortunella*. The different groups are represented with different colors. (C) Geographical information from 73 sequenced samples. The base layer of the map from Tianditu, the National Platform for Common Geospatial Information Services (NPCGIS) https://www.tianditu.gov.cn/. The base layer is under CC BY 4.0 license. (D)ROH analysis of the five groups. The y-axis indicates the length of the ROHs (Mb) in each accession. (E) Genetic statistics for the five groups. The heatmap indicates the pairwise differentiation (Fst) statistics. The genetic diversity (π) value of each group is shown. (F) Demographic histories of the five groups. The x-axis indicates generations. The y axis indicates the effective population size (*N*_e_). The estimated results from jackknife are indicated with translucent lines.

To investigate the population structure and demographic history of *Fortunella*, short-read sequencing from 73 accessions with approximately 30-fold coverage were collected, including 14 newly sequenced wild *F*. *hindsii* individuals (Fig 2C and S3 Table). The short reads from 15 accessions of the primitive citrus species (*Atalantia buxifolia*) were used as an outgroup in our genomic analysis. *Fortunella* samples were identified that perform two different types of reproduction (see Methods) [27]. For our purposes, we did not include accessions related by somatic mutations. These data were mapped to the Hongkong kumquat reference genome [27], resulting in a variation map that includes 10.03 million variations. The phylogeny, PCA analysis and ancestry composition estimation indicate that the cultivated and wild populations were separated into different clusters and that the divergent groups within the cultivated and wild populations were associated with distinct types of reproduction (Fig 2A and 2B). In particular, there are two major subpopulations in sexually reproducing populations (S9 Fig). Therefore, the *Fortunella* population was divided into five groups: cultivated apomicts (CULAPO), cultivated sexual (CULSEX), wild apomicts (WILDAPO), wild sexual subpopulation1 (WILDSEX1) and wild sexual subpopulation2 (WILDSEX2). We found a high level of differentiation (*F*_*st*_) between WILDSEX1 and WILDSEX2 groups (*F*_*st*_ = 0.1332) and that was striking higher than the differentiation compared with the WILDAPO group (e.g., the *F*_*st*_ between the WILDSEX1 and WILDAPO groups was 0.0619) (Figs 2E, S10, S11 and S12). In contrast to the broad dispersion of the apomicts, sexually reproducing samples were often found in small and isolated populations. Therefore, our results at least have the potential to confirm the fragmentation of wild sexually reproducing kumquats.

Estimates of effective population size are important for understanding the demography of endangered species [14]. We used SMC++ [35] analysis to investigate the population histories of the different groups. Notably, strong bottlenecks were detected in the WILDSEX1 group. The *Ne* declined to ~1,000 at 1,000 generations before present (Fig 2F). Populations with decreased *N*_e_ are prone to inbreeding because mating among close relatives becomes unavoidable in populations with finite sizes [36]. To examine the levels of inbreeding in the five groups, we tested the genome-wide runs of homozygosity (ROHs) (estimated region length >500 kb), genetic diversity (*π*) and *Tajima’s D* value in wild and cultivated kumquats (S13 and S14 Figs). We found the longest ROH length (average length of 31.8 Mb) and the lowest genetic diversity in the WILDSEX1 group (Fig 2D and 2E). Meanwhile, the higher effective population size in the WILDSEX2 group might explain the smaller ROH length relative to the WILDSEX1 group. Therefore, our findings reveal a small population size and high levels of inbreeding in wild kumquats. Interestingly, we found similar ROH levels, genetic diversity and heterozygosity in the WILDAPO, CULAPO and CULSEX groups, which may be related to asexual reproduction—apomixis or clonal propagation. But whether the domestication involved such genomic features in cultivars remains an open question.

### Extensive introgression from cultivated populations to wild populations

**Fig 3:**
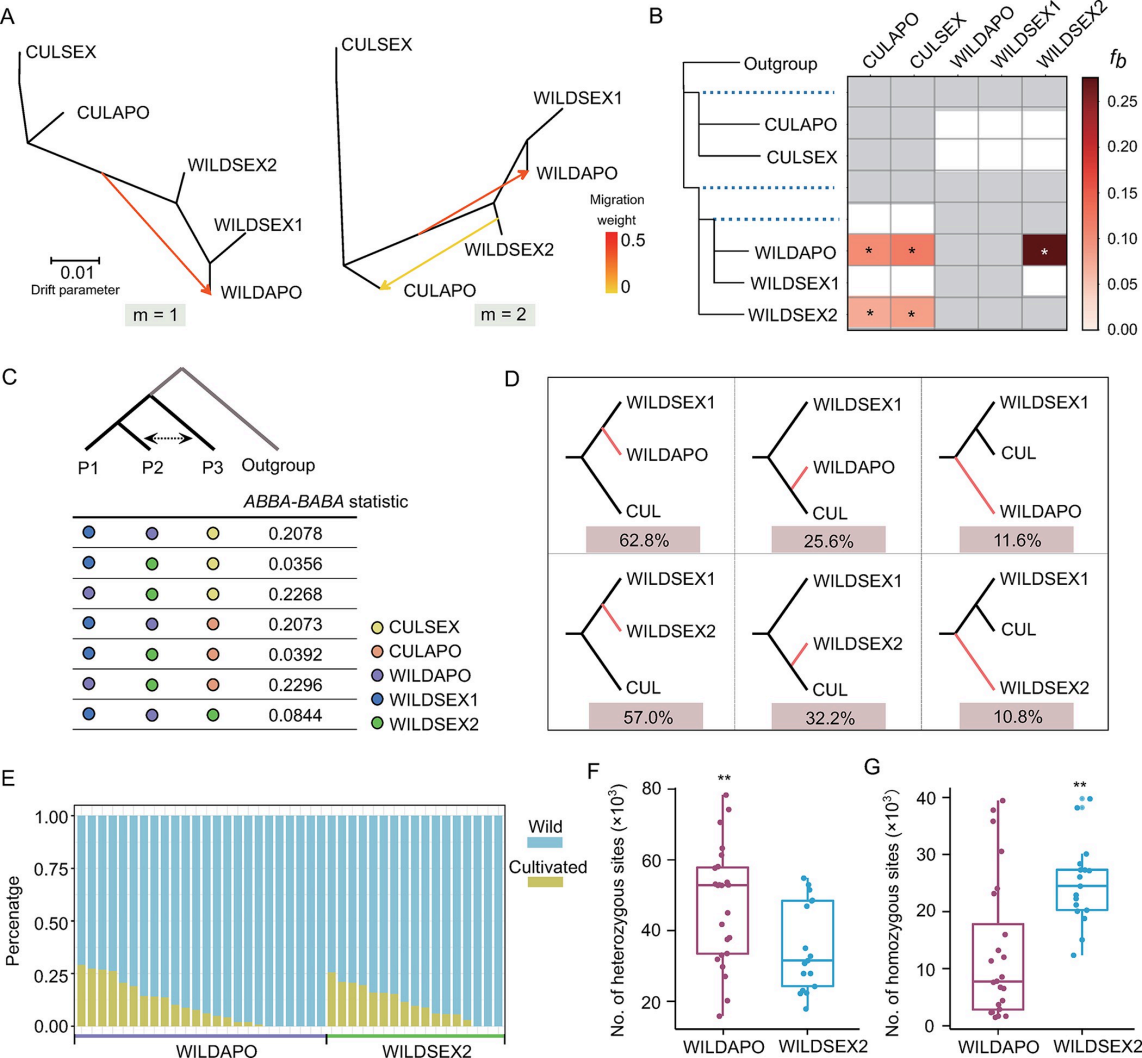
Extensive introgression from the domesticated population into the wild population. (A) Inferred graph and migration events based on genome-wide allele frequencies in the five groups. The directions are shown. The effect of migration events are indicated by the migration weight. (B) Heatmap of *f*_*b*_ statistics from the five groups. Significant *f*_*b*_ statistics are indicated with asterisks (*P* < 0.001). The 15 samples from *Atalantia buxifolia* were used as an outgroup. (C) Combinations of *ABBA-BABA* statistics and the corresponding values were calculated using the population-level data. (D) Topology weightings for two combinations (WILDSEX1, WILDAPO; CUL, Outgroup) and (WILDSEX1, WILDSEX2; CUL, Outgroup) used to estimate the introgression from cultivars in WILDAPO and WILDSEX2 groups, respectively. The CUL group is a combination of the CULAPO and CULSEX groups. (E) Proportion of introgressions from cultivars in samples from WILDAPO and WILDSEX2 groups. The proportions were calculated based on species-specific variations. (F) Number of heterozygous introgressed variations in WILDAPO and WILDSEX2 groups. (G) Number of homozygous introgressed variations in WILDAPO and WILDSEX2 groups. (F and G) **, *P* < 0.01 (Student t-test).

The ecological niche prediction suggests the potential for competition between wild and cultivated populations and the pattern of gene flow is unclear. Therefore, we inferred the graph and potential migration events based on the genome-wide allele frequency data. In addition to the simple bifurcating tree in *Fortunella*, our analysis inferred many admixtures between cultivated and wild populations (Figs 3A and S15). For example, the genotypes in the WILDAPO group could be traced from the ancestral populations of cultivars. It was also inferred that a migration event occurred between the WILDSEX2 and CULAPO groups (m = 2) that was associated with the lower migration weight. To obtain more evidence for candidate introgressions between cultivated and wild groups, we calculated a Patterson’s *D* statistic (i.e., an *ABBA-BABA* statistic) based on three groups (samples) comparisons and assuming that the outgroup was the primitive citrus *Atalantia buxfoliata* (Figs 3C and S16). The results revealed that significant signals from particular genotypes were shared between cultivars and wild samples (WILDAPO and WILDSEX2 groups). Although the *D* statistics revealed multiple significant gene flow events, it is not possible to exclude that a single gene flow event led to allele sharing in the different combinations of groups. Subsequently, we estimated the *f*_*b*_ statistic to quantify the potential correlated gene flow signals and branch-specific allele sharing patterns (see Methods). The significant *f*_*b*_ value in the internal branches indicates independent introgression events between the cultivar and wild groups (Fig 3B and S4 Table). On the other hand, our genome-wide analysis did not find any evidence of substantial genetic exchange between WILDSEX1 and the cultivated groups, possibly due to selection and genetic drift.

Noting the significant gene flow signals from different combinations of WILDAPO and WILDSEX2, we sought to estimate the proportion of shared alleles from cultivated populations. First, a topology weighting approach was used to explore the lack of coordination between local maximum likelihood (ML) trees and the species ML tree (see Methods). We found that 25.6% and 32.2% of the trees were uncoordinated in the WILDAPO and WILDSEX2 groups, consistent with shared alleles from cultivars. Second, we combined species-specific markers (SSMs), network phylogeny and genome-wide *f*_*d*_ statistics to identify the regions subjected to introgression (S17 and S18 Figs, see Methods). We found heterogeneity in the introgression ratios and genomic regions in the individuals from both WILDAPO and WILDSEX2 groups (Fig 3E). Some varieties acquired more than 25% of their alleles from cultivated populations through introgression (S5 Table). Furthermore, we calculated the number of heterozygous and homozygous variants from cultivated populations. We found that more introgressed variants appear to be heterozygous in the WILDAPO group than in the WILDSEX2 group. In contrast, there were fewer homozygous introgressed variants in the WILDAPO group (S19 Fig). As a possible explanation, the different reproductive types might influence the status of the introgressed alleles. Although the phylogenetic and allele frequency patterns among the groups were at least partially influenced by the incomplete lineage sorting (ILS), there is a general consensus based on multiple analyses. Collectively, our analyses support extensive introgression from cultivated populations to wild populations that reproduce sexually and through apomixis.

### Accumulation of genetic load in wild populations

**Fig 4:**
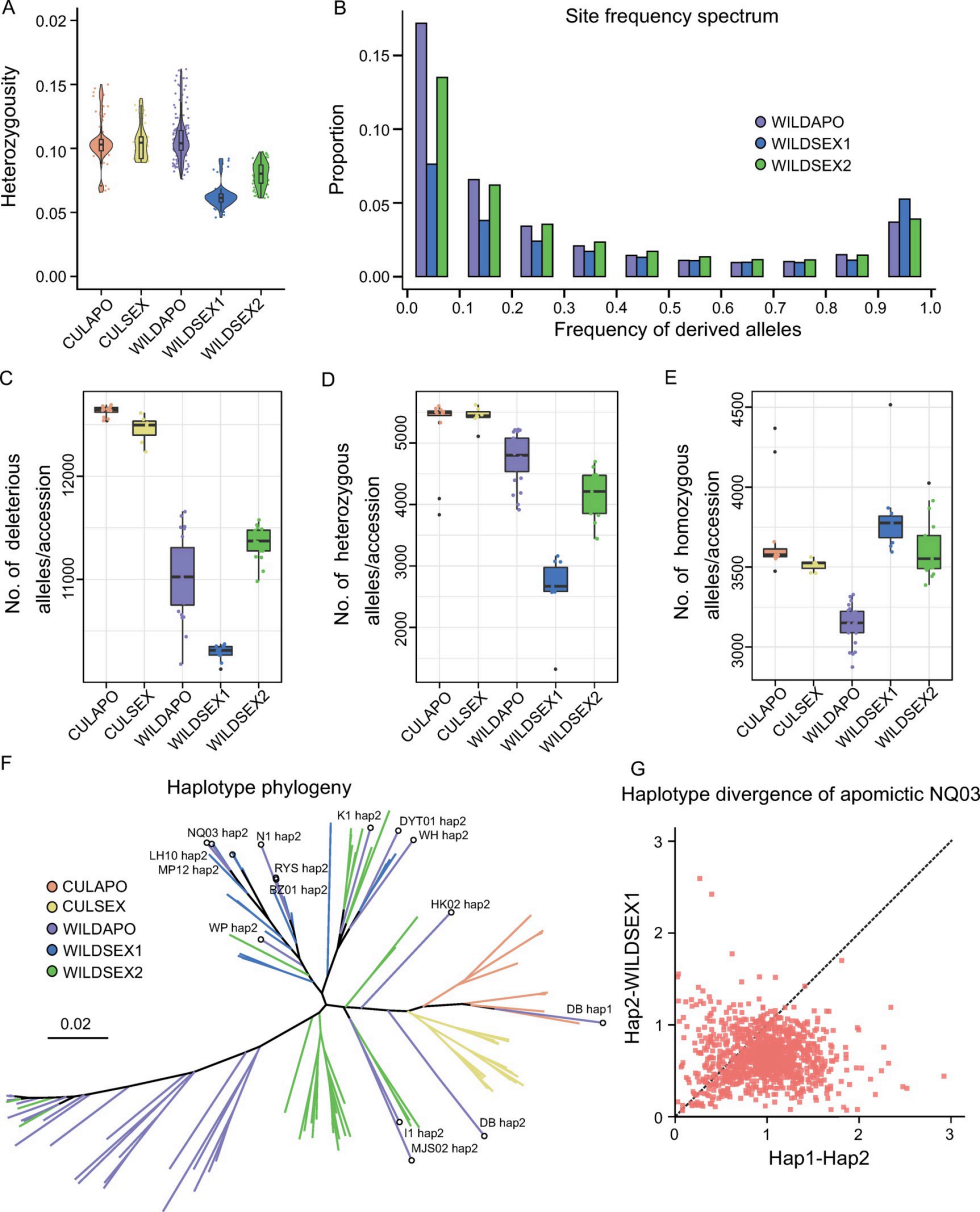
Genetic load of the wild population. (A) Heterozygosity of accessions in the five groups. (B) Unfolded site frequency spectrum (uSFS) of deleterious SNPs (dSNPs) in the three wild kumquat groups. (C) Number of deleterious mutations per accession in the five groups. ***, *P* < 0.001 (Student t-test). (D) Number of heterozygous deleterious mutations per accession. (E) Number of homozygous deleterious mutations per accession in the five groups. (F) Haplotype phylogeny of the apomixis region in 71 accessions from the five groups. (G) Standardized haplotype distance (divergence) of wild apomictic sample NQ03 using a 25-kb non-overlapping window. Hap2 indicate that the haplotype did not cluster with the WILDAPO group. Hap1 indicates the rest. The dotted line indicates that the distance between the haplotypes in NQ03 is equal to that between Hap2 and the haplotypes from the WILDSEX1 group.

To elucidate the demographic histories and selection patterns that drive the apomictic and sexually reproducing groups toward the most well adapted state, we estimated the genome heterozygosity of five groups. The results provide evidence that the apomictic group had higher rates of heterozygosity relative to the sexually reproducing wild groups (Fig 4A and S6 Table). Although the different reproductive types may influence the heterozygosity of wild kumquat groups, there was no significant differences between the sexually reproducing and apomictic groups from the cultivated population. These cultivars have experienced domestication and prolonged clonal propagation, which might explain the inconspicuous patterns between the different reproductive types.

Deleterious alleles were defined as variants that potentially determined fitness [37]. Therefore, we used genomic approaches to study the accumulation of genetic load among the different groups of endangered wild kumquat. In particular, the genome-wide putatively deleterious mutations were identified based on ancestral alleles (see Methods). The unfolded site frequency spectrum (uSFS) suggested that most deleterious mutations accumulated at minor frequencies in the WILDAPO group (Fig 4B). At the same time, the proportion of minor frequencies in the WILDSEX1 group was extremely low and the deleterious mutations were fixed at the highest rates, which might be influenced by genetic drift and a relaxed purifying selection (S20 Fig). In addition, we counted the number of deleterious mutations for the five groups in each accession. Our results showed that the number of deleterious mutations in the cultivated groups was significantly higher relative to the wild groups (Figs 4C and S21). As a result of this difference, introgression from cultivars might increase the hybridization load. In wild kumquats, we found that the number of heterozygous deleterious mutations was higher in the apomictic group and that the number of homozygous deleterious mutations was lower relative to the sexually reproducing groups (Fig 4D and 4E). As a possible explanation, the apomictic varieties could harbor hidden deleterious mutations in a heterozygous state. Collectively, our findings are consistent with different reproductive patterns shaping the genetic load patterns of wild kumquats.

Although the apomictic wild group accumulated the most heterozygosity and heterozygous deleterious mutations, the question remained whether apomicts prevent the loss of fitness caused by deleterious homozygous alleles in leaky sexual reproduction (S22 Fig). The haplotype tree derived from the apomixis determining locus (Chr4: 29.1–29.6 Mb) can easily trace the inheritance of haplotypes [27]. Therefore, we analyzed the haplotype tree of apomictic loci (see Methods). The phylogenetic analysis is consistent with a close association between one haplotype in some apomicts and another haplotype from the WILDSEX1 or WILDSEX2 groups (Fig 4F). In particular, both haplotypes from the apomictic variety DB were clustered with cultivated kumquats. Those results revealed that outcrossing in leaky sexual reproduction in apomicts is common and even occurs between different populations and species. Furthermore, we examined the genome-wide haplotype divergence in the WILDAPO group using a 25-kb window (S23 and S24 Figs, see Methods). For example, we found a higher haplotype divergence in 79.6% of the genomic regions in the apomictic variety NQ03 relative to the haplotypes in the WILDSEX1 group (Fig 4G). Collectively, our findings traced potential outcrossing events in apomictic wild kumquats in leaky sexual reproduction, which may avoid the accumulation of deleterious homozygous alleles.

### Influence of reproductive types on genomic heterozygosity in *F*. *hindsii*

**Fig 5:**
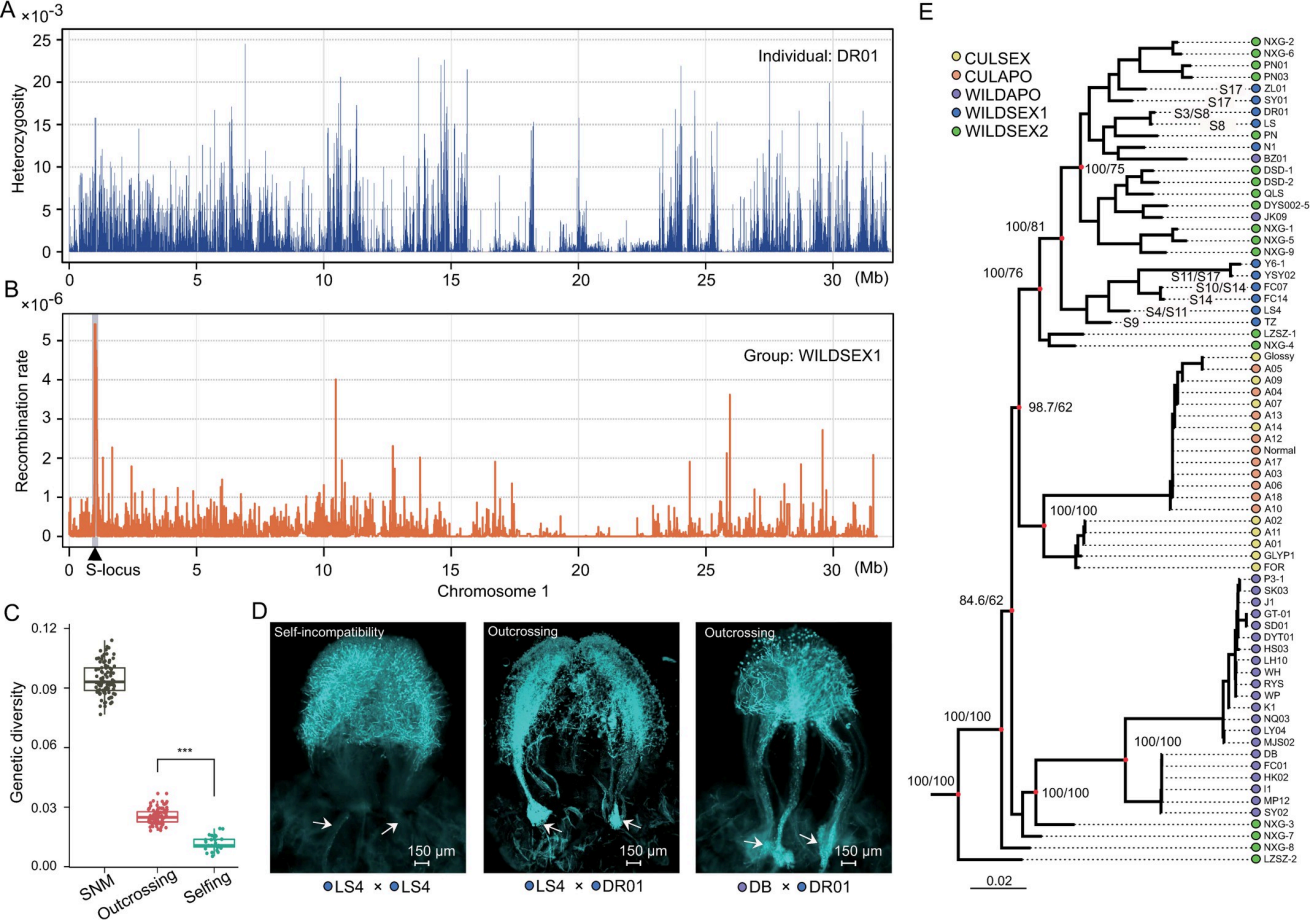
Self-incompatibility in the wild sexually reproducing population. (A) Pattern of heterozygous peaks for the sexually reproducing individual DR01 for Chr1. (B) Recombination rate of the WILDSEX1 group for Chr1 with the *S*-locus indicated with a triangle and gray shading. (C) Forward simulations of genetic diversity in two reproductive types compared to the standard neutral model (SNM). (D) Representative fluorescence images of aniline blue stained pistils five d after pollination. Data from two outcrosses and one self-cross are shown. The accession names are indicated (bottom). Scale bars = 150 μm. Vascular bundles are indicated with arrows. (E) Local tree of the *S*-locus (Chr1: 0.8–1.2 Mb). The tree was constructed and presented with bootstrap values. The eight identified *S*-genotypes in the WILDSEX1 group are shown.

Although self-fertilization is an important sexually reproducing strategy, many self-fertilizing lineages appear to be short-lived relative to related outcrossing lineages [38]. To explore reproduction in the sexually reproducing groups, especially the higher inbreeding levels in the WILDSEX1 group, we analyzed the genome-wide heterozygosity peaks. The results do not provide evidence for genomic flatlining in sexually reproducing individuals (Fig 5A). To elucidate the effects of outcrossing and selfing; we performed a forward simulation to analyze the genetic diversity of the two different reproductive types based on the demography of the WILDSEX1 group (S25 Fig, see Methods). We found that the genetic diversity in the outcrossing population was significantly lower (*p* < 0.01) than the standard neutral model (SNM) and was protected against loss of genetic diversity relative to the selfing population (Fig 5C).

Self-incompatibility (SI) is common in citrus [39]. SI promotes outcrossing and might explain the genome-wide heterozygous pattern in sexually reproducing kumquats. To test this hypothesis, we identified the phenotypes within the WILDSEX1 group. We found that all sexually reproducing individuals were self-incompatible (Fig 5D). In addition, our analysis indicates that the S-locus (Chr1: 0.95–1.15 Mb) had the highest population recombination rate for chromosome 1 in the WILDSEX1 group (Fig 5B). The balancing selection can overcome the effects of genetic drift and prevent allelic fixation, which may explain the high genetic diversity and recombination rate in the *S*-locus for populations that reproduce sexually [40]. Furthermore, we used ML trees to investigate the diversity of *S* alleles in *Fortunella* (Fig 5E). The results showed longer external branches in wild groups that reproduce sexually and at least eight genotypes that have been identified in the WILDSEX1 group (S7 Table and S26 Fig). In contrast, we observed much less diversity in the cultivated and apomictic individuals, which may be related to reductions in the efficiency of recombination or increased genetic drift due to apomixis or clonal propagation. However, it is not possible to exclude effects from domestication in cultivars [23]. Collectively, our analyses examined the importance of reproductive mechanisms in wild kumquats that perform sexual reproduction against the potential consequences of inbreeding.

## Discussion

### Genomics applied to the conservation of wild kumquat

**Fig 6:**
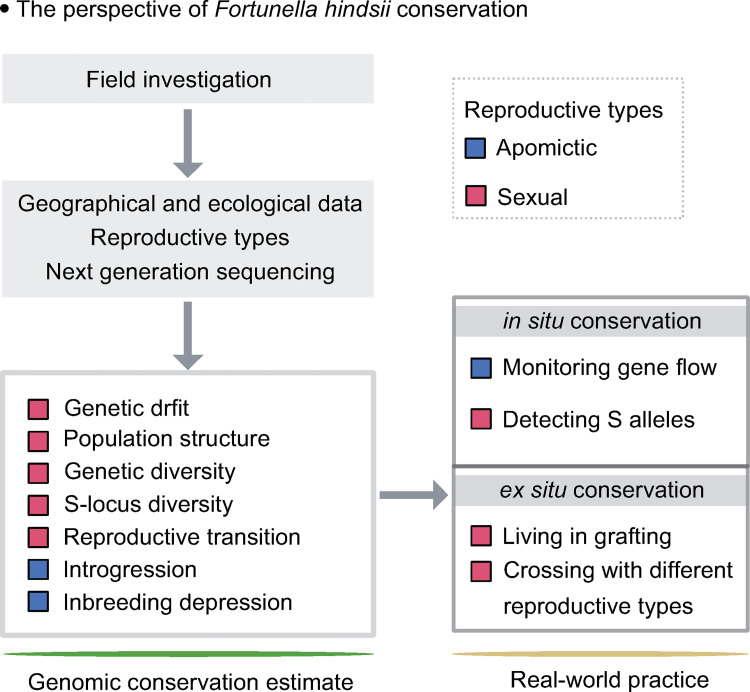
Conservation recommendations for endangered wild kumquats. The conservation strategy including a summary of the genomic conservation estimate and real-world practices for the endangered wild kumquats.

Our goal was to explore the ecological niche competition, high level of inbreeding and uncontrolled gene flow in the endangered wild kumquat. We found that decreased effective population size and high levels of inbreeding in wild kumquats that reproduce sexually because of population fragmentation (Fig 2). At the time, the ecological niche of cultivated kumquat overlapped with its wild relatives (Fig 1), thus human activities leading to the spread of cultivated varieties might contribute to extensive gene flow from the cultivated population into its wild population (Fig 3). In addition, the genomic landscape of introgression and genetic load were significantly influenced by reproductive patterns. Within apomictic kumquats, broad hybridization was encouraged and recombination rates were reduced, which led to more extensive regions of heterozygous introgression and deleterious mutations hidden by heterozygosity (Figs 3 and 4). Meanwhile, we found a significant increase in homozygous deleterious alleles in the small sexually reproducing group (Fig 4). Furthermore, our evaluation of the outcrossing mechanism of the SI system in the wild sexually reproducing population demonstrated that outcrossing may more effectively avoid genomic flatlining and extinction relative to selfing and thus, is consistent with previous work [41] (Fig 5). Finally, our genomic conclusions contribute to the conservation of genetic resources and provide concrete recommendations for conservation practices (Fig 6).

Access to genomic data has recently opened up new avenues for conservation research [42]. Our study focused on genomics-derived information relevant to the conservation of the wild relatives of citrus. In particular, we emphasize the utility of genomic data for two different aspects of conservation biology [43]: (i) understanding environmental adaptations, evolutionary histories and reproductive patterns and (ii) more specifically, describing the genetic load in the population and the introgression from the cultivated population [44]. Although it is difficult to rule out the effect of introgression, genomics tools provide the possibility of estimated evolutionary histories. For example, haplotype information is used to investigate inbreeding levels by identifying long tracts of the genome that are identical within and between individuals (Fig 2). Moreover, genetic statistics are helpful in identifying individuals harboring introgressed DNA and the genomic regions that were introgressed from the cultivated population (Fig 3). Accordingly, genomic approaches will help to resolve issues central to the conservation of wild relatives of crops.

### How do reproductive patterns inform conservation practices for wild relatives of citrus?

Natural selection, genetic drift, gene flow and reproductive patterns can shape the genetic load patterns in a finite population [45]. We demonstrated that the accumulation of deleterious mutations in the genome were influenced by reproductive patterns and area major concern for conserving the wild relatives of citrus [46]. We found that the introgressed regions were mostly heterozygous and that the deleterious mutations were hidden in the heterozygous state in the wild apomicts (Figs 3 and 4). There are two possible explanations for these findings: 1) a reduction in the recombination rate due to apomixis contributed to the reduced efficiency of natural selection [47]. In this scenario, uncontrolled gene flow from the cultivated population into wild apomictic populations led to a relaxation of selection and the retention of introgressed regions [12]. 2) extensive outcrossing with the sister populations that reproduce sexually or with non-related apomictic varieties to avoid the accumulation of homozygous deleterious alleles [48]. Given that the highest heterozygosity and genetic diversity were observed in the apomictic populations, the effects of leaky sexual reproduction cannot be ignored [49]. Besides, the accumulation of genetic load was strongly influenced by genetic drift in small populations of the sexually reproducing kumquat [14]. For example, we observed a higher number of homozygous deleterious mutations in the WILDSEX1 group (Fig 4). Although inbreeding increases the homozygosity of offspring and thus, may lead to the purging of exposed homozygous deleterious alleles, this mechanism does not appear to counteract the effects of genetic drift in isolated populations of wild kumquat [15,50]. Collectively, our data indicate that reproductive patterns are critical for the genomic conservation of endangered populations of wild relatives of citrus.

Genomic flatlining refers to the reduced genetic diversity of endangered species and obviously depends on population size and selfing rates [51]. Genomic flatlining is a potential cause of the decreased adaptation of selfing populations and can be estimated by genome-wide variations as selfing populations become progressively unable to generate new genetic variability through recombination [52]. Our genomic data provide evidence that the SI system can preclude selfing and eliminate the concomitant effects of selfing (Fig 5) [53] and that the diversity of S-alleles serves a crucial role in fertilization for population reproduction [54].

### Conservation practices for *Fortunella hindsii*

The genetic resources from isolated and small populations are crucial for developing conservation practices for wild species. Fragmentation leads to population subdivision patterns that pose a crisis for the conservation of sexually reproducing kumquats [55]. Therefore, conservation genomic estimates and real-world practices were focused on genetic diversity, demographic histories, homozygous deleterious alleles and *S*-locus polymorphisms (Fig 6). In contrast, apomictic populations require more attention on introgression from cultivated populations. The contribution of heterozygous introgression and the genome-wide heterozygous deleterious mutations were quantified for insight into the possibility of genetic swamping from uncontrolled gene flow [56,57]. Thus, although genomics provides prioritized recommendations for conservation practices that should be implemented, conservation measures should be constructed based on reproductive patterns.

Gene rescue is a protective strategy that reduces the risk of extinction by increasing the absolute fitness of populations and is still controversial [58,59]. The debate focuses on whether the translocation of individuals or alleles into small, endangered populations will produce the desired effect [58]. The widespread use of outcrossing to genetically rescue inbred populations might induce outbreeding depression [60]. Therefore, genetic rescue as a conservation technique for historically small populations should be used with caution [61]. We report two reproductive modes in wild citrus populations. If genetic rescue is utilized in a wild sexually reproducing population, a reproductive pattern transition may occur. Apomixis is genetically controlled and inherited as a dominant trait. Therefore, the transition will hinder the recovery of sexual reproduction [62].

Although the conservation of wild kumquats began two decades ago, the details of the genomic patterns have only recently been revealed. Our previous efforts involved collecting samples for ex situ conservation (i.e., botanical garden, DNA storage and pollen storage) based on geographical information and classification based on the reproductive systems. Based on our conclusions from genomic data, we propose to set up conservation management units based on the reproductive patterns [63]. To prevent continued declines in fitness in the sexually reproducing subpopulations that we investigated, ex situ conservation of wild samples and alleles might include: (a) generation of complexes that allow hybridization between the subpopulations and (b) reintroduction of sexually reproducing offspring from artificial interspecific crosses performed with apomictic and sexually reproducing samples. Additionally, if diversity at the self-incompatibility locus constrains pollination activities and increases the risk of extinction, monitoring of the SI system should be considered.

## Methods

### Materials and reproductive phenotypes

Apomixis contributed to the offspring with identical genotypes as the maternal lineage. Therefore, the wild apomictic samples derived from the nuclear adventitious embryonic pathway were excluded from our conservation genomic analyses. Only one sample was maintained from each somatic lineage. Therefore, a total of 73 diploid samples were collected to represent the diversity of *Fortunella*. This included 19 accessions of cultivated kumquats and 54 accessions of wild kumquats. There are two different reproductive types in wild *Fortunella* samples. The cultivars (7 sexual and 12 apomictic varieties) were collected from five major production areas and related provinces including Guangxi, Hunan, Jiangxi, Fujian and Zhejiang.

Apomictic samples of wild kumquats were distributed over a broad geographic range. In contrast, the sexually reproducing wild samples occurred as small and isolated populations. To ensure representative genetic diversity in local areas, we collected sexually reproducing samples with a radius of at least 500m during the field investigation. For apomicts, collected 1–2 samples in each local region to avoid somatic samples. The 24 apomictic wild kumquats were identified and collected from Guangdong, Jiangxi, Hunan, Fujian and Zhejiang Provinces [27,28]. The 30 sexually reproducing wild kumquats were identified and collected from Guangdong and Fujian Provinces including the 14 newly sequenced diploid samples from Shenzhen, Guangdong Province. Previously published data from wild and cultivated kumquats were obtained from the National Center for Biotechnology Information (NCBI). The reproductive phenotypes were first reported by Wang et al. [27]. Meanwhile, the phenotypes of newly sequenced samples were identified based on the methods described by Wang et al. [64]. The previously collected wild kumquats had been grafted at the National Citrus Breeding Center at Huazhong Agricultural University (Wuhan, China). Our analysis of kumquats that reproduce sexually identified two recent changes in the type of reproduction in samples BLS07 and LT01 (see below) that were excluded from the conservation genomic analysis.

In addition, *Atalantia buxifolia* is a kind of primitive citrus. A total of 15 previously published short read sequences were downloaded from the NCBI database. Those reads were used as an outgroup in this study. The outgroup genotypes were used in phylogenies, introgression statistics and genetic load analyses. A phylogeny of 13 citrus species was constructed based on the variation map generated by Wang et al. [27] (https://zenodo.org/record/574866 2#.Y3YUqcdBxaY).

### Geographical distribution and ecological niche modeling

We collected 396 distribution records for *Fortunella* from the Chinese Virtual Herbarium (https://www.cvh.ac.cn/). The earliest collection was recorded in 1935. The original and complete records were maintained including records for 244 wild and 152 cultivated samples (S1 Table). The geographical information was used for the ecological niche analyses. Subsequently, the environmental data were collected with a spatial resolution of 2.5′ environment layers including 19 climatic variables (https://worldclim.org/data/bioclim.html). All 19 variables were used for a principal component analysis (PCA) of the 396 sites of cultivated and wild *Fortunella*. In addition, we used least absolute shrinkage and selection operator (LASSO) regression to select variables that are the most informative with respect to the distribution of wild and cultivated populations using the Glmnet v4.1.4 package in R [65]. The generalized linear model with penalized maximum likelihood was used to fit the prediction of climatic variables and species. Furthermore, the most informative climatic variables were selected. On the other hand, the 396 sites and the 19 associated environmental variables were used to analyze the niche overlap between the wild and domesticated populations using nicheROVER [66]. Finally, we predicted the distributions of cultivated and wild populations using two methods.1) We made predictions using BIOMOD2 [67] with three different models—General Linear Model (GLM), Generalized Boosting Model (GBM) and Random Forest (RF). 2) We independently made predictions using MAXENT [68] with 100 iterations.

### Genome sequencing and variation map construction

Whole genomic DNA from 14 newly collected wild kumquats was extracted from fresh young leaves. The quality of the DNA was checked using pulsed-field gel electrophoresis. Approximately 12 Gb (30-fold coverage) of paired-end short reads were generated for each sample using the Illumina NovaSeq 6000 platform. The short reads of 73 accessions from *Fortunella* and 15 accessions from *Atalantia buxifolia* were mapped to the Hongkong kumquat chromosome-level reference genome using BWA-MEM v0.7.17 (https://github.com/lh3/bwa). Subsequently, we removed the adapter sequences using the Fastp program [69]. The sequence alignment and map (SAM) files were sorted after the removal of PCR duplicates using Samtools v1.15 [70] and Picard 2.19.0 (https://github.com/broadinstitute/picard). Finally, genotype information was obtained using binary alignment and map (BAM) files with Deepvariant (rc1.0.0) and the default settings [71]. The 88 independent variant call format (VCF) files were consolidated into a single file using GLnexus (v1.2.7) [72]. To obtain reliable variations, we filtered nuclear genomic variations based on the depth of coverage and missing rates using VCFtools with the following criteria: variant quality (QD) > 2.0, quality score (QUAL) > 40.0, mapping quality (MQ) > 30.0, genotype calls with a depth > 2 or <100 and with < 20% of genotypes missing across all samples. Finally, approximately 10.03 million variations were identified in 73 accessions from *Fortunella* samples for downstream analyses.

### Population structure and genetic statistics

The variation map was first used to investigate population structure based on a maximum-likelihood (ML) phylogenetic tree, PCA analysis and ancestry composition estimation using SNPs from the entire genome. A ML phylogeny was constructed using IQ-TREE version 2.0 [73] with 1000 ultrafast bootstrap replicates that yield support values for each node using the ‘GTR + I+G’ model. The tree layout was generated using Figtree (http://tree.bio.ed.ac.uk/software/figtree). The PCA analysis was performed using the PLINK program [74]. Then, the variation map was used to investigate population structure using Admixture [75], evaluating each possible number of distinct groups, *K*, from 2 to 5 with fivefold cross-validation (—cv  =  5). Notably, genotype information from the outgroup was used only as a root in the phylogeny. In contrast, the PCA analysis and ancestry composition estimation excluded outgroup samples. The consensus results from those analyses were used to infer the population structure in the *Fortunella* genus. Our analysis identified two accessions that recently changed their mode of reproduction, BLS07 and LT01. These two accessions were highlighted and excluded from the downstream analyses. Finally, we defined five groups (CULAPO, CULSEX, WILDAPO, WILDSEX1 and WILDSEX2) that include 71 accessions of *Fortunella* based on population structure. Finally, the linkage disequilibrium (LD) decay of each group was determined using the variation map.

Furthermore, we calculated the nucleotide diversity (π) based on a 25-kb window (examined by LD decay) in each group using the Python script popgenWindows.py described by Martin et al. [76]. The pairwise genetic statistics, such as allele frequency differentiation (*F*_st_) and divergence (*D*_xy_), were also investigated using this Python script [76]. Meanwhile, *Tajima’s D* value was circulated using 100-kb windows in each group using the VCFtools program [77].

The genome-wide runs of homozygosity (ROHs) were detected using PLINK [74] with the following options: a minimum of 50 SNPs per ROH, at least 10 SNPs per 100 kb, a scanning window of 50 SNPs, a total length > 500 kb, spacing between successive SNPs < 100 kb and no more than three heterozygous SNPs allowed in each scanning window. The ROH length in each sample was calculated by summing all the detected ROHs.

### Inference of demography, heterozygosity and recombination

SMC++ [35] was used to infer the demographic history of the five groups from the cultivated and wild populations. A mutation rate of 2.2 × 10^−8^ per site [64] per generation was used for *Fortunella*. Genome regions were masked when the coverage depth was < 15 after reads with mapping quality < 20 removed to improve reliability. We phased the variations in the diploid genomes to obtain haplotype information using Beagle v5.0 [78]. Furthermore, we split the phased VCF into nine chromosomes and estimated demographic history separately for each chromosome. The mutation rate was used as described by Wang et al. [64]. The results from nine chromosomes were combined to infer the demography of the population. A jackknife procedure with 20 replications was used to verify the results using a 5 Mb region.

The recombination rate was estimated using the demography generated from SMC++ and Pyrho [79]. Our analysis generated five demographic history records for each group, including the effective population size and the corresponding breakpoint generation. To estimate the population recombination rate in each group, we set a larger sample size (N) for the approximate lookup table and a downsample size (n) for the test group. The downsample size could be the number of haplotypes in each group. The larger sample size could be smaller than twice the size of the downsample. For example, we estimated the recombination rate in the WILDSEX1 population using 11 individuals with n = 22 and N = 40. The recombination rates of the nine chromosomes from each group could be evaluated based on the same approximate lookup table.

We first estimated heterozygosity using the k-mers frequency method and the deep sequencing data (average 30-fold coverage) from 71 accessions of Fortunella. We counted the occurrences of k-mers based on the paired reads with k-mer sizes of 17, 19, 21, 23, 25 and 27 using Jellyfish v2.3.0 [80]. The k-mer counts were recorded in a binary format file. These files were used to estimate genome characteristics, such as genome size, heterozygosity, and repetitiveness using the GenomeScope v2.0 program [81]. We examined the genome size and repetitiveness to exclude overfitting for each estimation. There were nine chromosomes in the collected diploid samples, while the genome sizes were ~330 Mb based on kmers. The genome heterozygosity of each sample was generated for the downstream analysis. Second, we estimated the F(het) value using the PLINK program and the variation map with default parameters. To gain insight into the genetic mechanism that prevents genomic flatlining in the wild sexually reproducing population, we calculated the heterozygous peaks at the chromosome level using non-overlapping 100-kb windows and a custom script.

### Introgression analysis

The graph and the migration of the five groups from *Fortunella* were constructed using a variation map created using Treemix v1.11 [82]. We prepared the allele frequencies from the five groups and generated a ML tree for the five groups based on five repeats. To estimate the primary or highest possible introgression among the five groups, we estimated that the potential migration events ranged from 1 to 3 with five repeats for each test. Subsequently, the migration edges and directions were highlighted and added to the graph using the plotting_func.R script.

In addition to estimating introgression among the five groups based on allele frequencies, we incorporated Patterson’s *D* statistic (i.e., the ABBA-BABA statistic) to examine gene flow between the test groups [83]. We detected the potential signals of introgression between P2 and P3 using the different triplets. The genotype information from 15 *Atalantia buxifolia* samples were used as the outgroup to test the topology (i.e., P1, P2; P3, Outgroup). The genotypes shared between P2 and P3 were detected, and the statistics were analyzed using a jackknife. To obtain reliable results, different populations were considered as the P1 group. The extensive introgression between the wild (WILDAPO and WILDSEX2) and cultivated populations (CULAPO and CULSEX) were analyzed in six combinations. A topology analysis (WILDSEX1, WILDAPO; WILDSEX2, Outgroup) was performed to find the most shared homozygous genotype between the WILDAPO and WILDSEX2 groups. In addition, we estimated the *D* statistic based on BAM files at the individual level using the ANGSD program [84]. The topology of individuals is the same as the combination of *D* statistics at the population-level. Although the *D* statistic contributes to the potential migration events between the two test groups, the combination of sister clades appeared to discriminate between single events and independent migration events in related clusters. Therefore, we calculated the *f*_*b*_ statistic using the Dsuite program and the topology generated from allele frequencies based on whole genome SNPs [85]. The *f*_*b*_ value indicates the proportion of shared genotypes between the two test groups. The potentially independent migration events were detected in the internal clade of wild populations. The *f*_*b*_ value calculated using WILDAPO and WILDSEX2 indicated that they were the most similar genotypes and was used as a positive control.

The monophyletic nature of the five groups results from extensive introgression and hybridization and creates difficulties when trying to represent the evolutionary history of the five groups. To resolve this problem, we used the topology weightings method to examine the incoordination between the genome-wide phylogenetic tree and the local tree using Twisst (https://github.com/simonhmartin/twisst). Genome-wide subtrees were generated using a 25-kb window and IQ-TREE version 2.0 with 1000 ultrafast bootstrap replicates and the ‘GTR + I+G’ model. To obtain reliable results, we kept the subtrees with more than 500 variations in each window. Our analyses are consistent with sexually reproducing and apomictic cultivars contributing to the WILDAPO and WILDSEX2 groups. Therefore, we created a CUL group by merging the CULAPO and CULSEX samples to detect uncoordinated topologies from comparisons of the wild and cultivated populations. In addition, there were no introgressed signals in the sexually reproducing WILDSEX1 group. The two combinations (WILDSEX1, WILDAPO; CUL, Outgroup) and (WILDSEX1, WILDSEX2; CUL, Outgroup) were also analyzed to investigate genome-wide *f*_*d*_ statistics using a 25-kb window [76]. The supported topologies and corresponding proportions were plotted.

Finally, we sought to identify the potentially introgressed regions and introgressed proportions for each individual in WILDAPO and WILDSEX2 groups. Therefore, we identified species-specific markers (SSMs) to distinguish cultivated and wild populations as described by Wu et al. [86]. We identified variants fixed in cultivated (CUL) and the wild sexually reproducing (WILDSEX1) populations that retained different homozygous variations in the two groups. To reduce the influence of ILS, we used the outgroup genotype information to filter the unfixed variants. Thus, the final SSMs and the corresponding genotypes were used to calculate the proportion of introgression for each individual in WILDAPO and WILDSEX2 using the Admixture program [75,87]. Both heterozygous and homozygous introgressed sites were counted to investigate patterns of introgression in the wild sexual and apomictic kumquats using the custom script. In addition, the network phylogeny was constructed using Split-Tree [88] based on the SSM sites.

### Estimation of genetic load

We estimated the genome-wide genetic load as the number of deleterious mutations in the genome [89]. The gene structure and annotation file of the reference genome was used to predict potential deleterious mutations with the SIFT 4G algorithm [90]. To reduce the bias of reference as described by Wang et al. [27], we inferred the ancestral genotypes using the *Atalantia buxifolia* samples. We created genomic databases using the SIFT 4G Annotator (https://github.com/paulineng/SIFT4G_Create_Genomic_DB). Based on the SIFT annotation database, an amino acid substitution was predicted to be deleterious if the score was ≤0.05 and tolerated if the score was >0.05. We counted the number of deleterious, heterozygous and homozygous deleterious alleles in each individual in the five groups as recommended by Zhou et al. [89]. However, we cannot exclude the reference bias in the functional classifications for SIFT prediction [91]. In addition, the total number of derived deleterious alleles per individual equals the number of sites containing derived (segregating sites + 2*fixed sites) variants within that taxon [50]. Because of the unclear selective pattern; however, this method cannot exclude that some fixed ancestry alleles might reduce the fitness of individuals or groups [92].

The site frequency spectrum (SFS) was calculated based on the deleterious mutation dataset. Our primary aim was to study the conservation of wild Fortunella and to determine whether domestication might influence the SFS pattern of cultivated populations. Therefore, we compared the SFS patterns of deleterious mutations in the three groups (WILDAPO, WILDSEX1 and WILDSEX2) to study the effects of reproductive patterns in wild kumquats. The frequencies of each group were classified into ten clusters, excluding the reference (frequency of derived alleles > 0), using the easySFS program (https://github.com/isaacovercast/easySFS).

### Haplotype analysis

To evaluate the dominance of outcrossing in the leaky sexual reproduction of apomicts that prevents the accumulation of homozygous deleterious alleles, we performed haplotype analyses using two methods: 1) haplotype phylogeny of local ML trees (apomictic determining region) constructed with 71 accessions from the five groups and 2) the haplotype divergence analysis of the two haplotypes in each individual in the WILDAPO group.

The genomic region that determines apomixis was identified by Wang et al. [27]. Given that leaky reproduction rarely restores apomicts to strict sexual reproduction, a local tree constructed from the apomictic region can provide more information on haplotypes in apomictic samples in the WILDAPO group. Using a custom script, we split the haplotypic information based on the phased variation map. Subsequently, the phased haplotypic VCF format file of the apomictic region was converted to the PHYLIP format using the vcf2phylip program (https://github.com/edgardomortiz/vcf2phylip). Then, we constructed the haplotype phylogeny of the apomictic region using IQ-TREE version 2.0 with 1000 ultrafast bootstrap replicates and the ‘GTR + I+G’ model. The haplotypes that did not cluster with the WILDAPO group were highlighted and named hap2. Neither haplotype from the wild apomictic variety DB clustered with WILDAPO. These haplotypes were highlighted and named DB hap1 and DB hap2.

Besides, we calculated the haplotype divergence of each apomictic wild sample using a 25-kb window using two steps. 1) We prepared the phased haplotypic VCF file for each window. 2) We calculated the genetic distance of each haplotype using the VCF2Dis program. Therefore, the genetic distance represents the divergence of each haplotype in the window. To compare the distances of the windows, we normalized the genetic distance by introducing the mean value of each window as a background. For example, the value of NQ03 Hap1-Hap2 represents the normalized distance between the two haplotypes of the apomictic variety NQ03 in each window. On the other hand, the value of Hap2-WILDSEX1 represents the average distance between Hap2 (i.e., the haplotype that did not cluster with the WILDAPO group) from NQ03 and the haplotypes from the samples in WILDSEX1, which was calculated to evaluate the possibility of outcrossing during leaky sexual reproduction in the apomictic variety NQ03.

### Characterization of self-incompatibility

To characterize the outcrossing mechanism in the wild sexually reproducing kumquat, we tested for self-incompatibility in the 13 wild sexually reproducing varieties including nine samples from the WILDSEX1 group and four samples from the WILDSEX2 group (QLS, PN, PN01 and PN03). Additionally, a cross-pollination experiment was performed with the apomictic variety DB and the sexually reproducing varieties. The cross-pollination and self-pollination experiments were performed 1 d before anthesis. Five d after pollination, pistils were excised and fixed in a mixture of alcohol and acetic acid (4:1). The growth of the pollen tubes within the style was observed using the aniline blue fluorescence staining method. These samples were maintained in a garden by grafting and thus, the self-pollination experiments could be strictly controlled. These newly collected samples have yet to bloom. In addition, 17 known alleles of S-RNase genes in citrus were identified previously [39]. To identify candidate S-RNases involved in self-incompatibility and to characterize the diversity of S alleles, short reads from 13 wild sexually reproducing samples were mapped to the sequences encoding *S-RNase*s using the BWA-MEM program. Using this alignment, the eight S-RNase genotypes reported for citrus were identified in the WILDSEX1 kumquat population.

The pollination experiments have been performed only in four samples from the WILDSEX2 group. Therefore, to obtain a big picture for the pattern of self-incompatibility in Fortunella, we constructed a local phylogeny to investigate the genetic features of the S-locus using IQ-TREE version 2.0 with 1000 ultrafast bootstrap replicates and the ‘GTR + I+G’ model. A total of 71 accessions from five groups were used to construct the phylogenetic tree for the *S*-locus. The *S*-locus has been undergone balancing selection [40]. Our reference genome contained only one type of S locus; therefore, we constructed phylogeny using fixed polymorphisms.

### Forward simulations with different reproductive types

We used the SLiM [93] software (version 3.2.0) to perform forward simulations and obtained 100 replicates using a demographic model based on the wild sexually reproducing species (i.e., the WILDSEX1 group) inferred with SMC++. We simulated a population of *N* = 1,000 individuals that were run for 10*N generations to reach an equilibrium. We then introduced a 0.25*N size bottleneck at generation 10,000 until generation 2,000. For the subpopulations, we assumed two types of mating systems: outcrossing and selfing. We introduced mutations into the simulation that could have one of three different effects on fitness based on the simulated genomic region. (1) ‘Neutral’: all mutations within the coding region are neutral (*s* = 0). (2) ‘Deleterious’: recessive mutations that are deleterious are present in the populations. The effect on fitness is drawn from a gamma distribution (DFE) [94] (dominance coefficient, *h* = 0). All simulated genomic regions had a length of 1 Mb. We used the length of the exons from chromosome 1 in the kumquat reference genome. The per base pair mutation rate was fixed at 2.2e-8 [64] and the recombination rate was fixed at 1.0e-7 [95]. The output records were generated using the SLiM program. The data were converted to VCF format files using a custom script. Finally, the genetic diversity (π) was calculated using the python script popgenWindows.py.

**Copyright:** This is an open access article distributed under the terms of the Creative Commons Attribution License, which permits unrestricted use, distribution, and reproduction in any medium, provided the original author and source are credited.

## Ethics approval and consent to participate

The plants collected in this study comply with the IUCN Policy Statement on Research Involving Species at Risk of Extinction and the Convention on the Trade in Endangered Species of Wild Fauna and Flora.

## Supporting information

S1 Fig Pictures of wild kumquats (*F*. *hindsii*) and its native environments. The wild kumquats are mostly grown on hillsides, with low distribution density.
(TIF)

S2 Fig Pictures of cultivated kumquats (*F*. *crassifolia or F*. *japonica*) and its plantations. The cultivated kumquat is a kind of intensive farming with uniform genotype by grafting. There are a series of agricultural technologies in this process.
(TIF)

S3 Fig The correlation of 19 bioclimatic variables based on 396 geographical data. Bioclimatic variables were derived from the monthly temperature and rainfall values to generate more biologically meaningful variables. The bioclimatic variables represented annual trends, seasonality and extreme or limiting environmental factors. The bioclimatic matrix was generated based on the 396 geographical data of Fortunella genus.
(TIF)

S4 Fig The principal component analysis (PCA) based on the bioclimatic matrix from *Fortunella* genus. The PCA analysis was constructed based on bioclimatic matrix of the 396 geographical data and used to investigate the most informative variables related to the distribution of wild and cultivated samples. PC1 represented the first principal component with interpretation of 47.5% variations. PC2 represented the second principal component with interpretation of 20.6% variations. The wild and cultivated populations were distinguished with different color. Each characteristic influences a principal component is highlighted.
(TIF)

S5 Fig The niche overlap probability of wild and cultivated populations in *Fortunella* using nicheROVER. Niche overlap was calculated as the probability that an individual from species A is found in the niche region of species B. The niche regions and pairwise niche overlap of wild and cultivated populations in *Fortunella* were calculated using 19 environmental variables. (A) The distribution of overlap probability that an individual from cultivated population was found in the niche region of the wild population. (B) The distribution of overlap probability that an individual from wild population was found in the niche region of domesticated population. The solid line showed the average of niche overlap probability and the dashed lines showed 95% probability of niche overlap probability.
(TIF)

S6 Fig The prediction of most informative climatic variables for distribution of wild and cultivated populations based on least absolute shrinkage and selection operator (LASSO) regression. The bioclimatic matrix of *Fortunella* genus was used to predict using the Glmnet program. (A) The cross-validated fit for selected values of lambda (log scale). (B) The coefficients curve corresponds to variables were plotted. The best fitting was highlighted. The y-axis indicated the number of coefficients at the current lambda.
(TIF)

S7 Fig The predicted distribution of wild and cultivated populations using BIOMOD2. The 19 bioclimatic variables were used for distribution modeling. The heatmap showed the predicted distribution of wild and domesticated populations using Generally Linear Model (GLM) and Generalized Boosting Model (GBM) models, respectively. The color presented the probability of prediction, and the resolution is 5 min. The base layer of the map from Tianditu, the National Platform for Common Geospatial Information Services (NPCGIS) https://www.tianditu.gov.cn/. The base layer is under CC BY 4.0 license.
(TIF)

S8 Fig The predicted distribution of wild and domesticated populations in *Fortunella* using MAXENT. The heatmap showed the predicted distribution with default parameters and generated with 100 iterations. (A) The predicted distribution of wild kumquats. (B) The predicted distribution of cultivated kumquats. The base layer of the map from Tianditu, the National Platform for Common Geospatial Information Services (NPCGIS) https://www.tianditu.gov.cn/. The base layer is under CC BY 4.0 license.
(TIF)

S9 Fig The Gene identity by descent (IBD) analysis of 54 sequenced samples from wild *Fortunella*. The pairwise IBD values were calculated based on genome-wide variations of the 54 sequenced samples in *Fortunella* using PLINK. The samples from different groups were distinguished with different color.
(TIF)

S10 Fig The statistics of genome-wide genetic diversity are calculated in three wild groups. The genome-wide genetic diversity (*π*) of the WILDAPO, WILDSEX1 and WILDSEX2 groups were calculated based on the variation map. Those statistics were calculated based on 25 kb non-overlapping windows.
(TIF)

S11 Fig The pair wise statistics of genome-wide differentiation (Fst) are calculated in three wild groups. The genome-wide differentiation (Fst) was performed between three combinations: between WILDAPO and WILDSEX1 groups, between WILDAPO and WILDSEX2 groups, and between WILDSEX1 and WILDSEX2 groups. Those statistics were calculated based on 25 kb non-overlapping windows.
(TIF)

S12 Fig The pair wise statistics of genome-wide divergence (Dxy) are calculated in three wild groups. The genome-wide divergence (Dxy) was performed between three combinations: between WILDAPO and WILDSEX1 groups, between WILDAPO and WILDSEX2 groups, and between WILDSEX1 and WILDSEX2 groups. Those statistics were calculated based on 25 kb non-overlapping windows.
(TIF)

S13 Fig The genome-wide ROHs analysis in five groups of *Fortunella*. The x-axis indicated the number of ROHs, while the y-axis indicates the length of ROHs in the genome. The samples from different groups were distinguished with different color.
(TIF)

S14 Fig The statistics of genome-wide *Tajima’s D* are calculated in five group *Fortunella* groups. The genome-wide *Tajima’s D* of the CULAPO, CULSEX, WILDAPO, WILDSEX1 and WILDSEX2 groups are calculated based on the variation map. Those statistics were calculated based on 25 kb non-overlapping windows.
(TIF)

S15 Fig The potential graph and three migration events were inferred based on the allele frequencies. (A) The allele frequencies of five groups were used to estimate the graph structure and the potential introgression events with the parameter m = 3. (B) The pair wise residuals of the graph modeling were calculated based on the genome-wide allele frequencies in five groups.
(TIF)

S16 Fig The *ABBA-BABA* statistics of 48 wild samples from the WILDAPO and WILDSEX2 groups. The D statistic was performed based on combinations of (WILDSEX1, WILDAPO; CUL, Outgroup) or (WILDSEX1, WILDSEX2; CUL, Outgroup) at individual-level using the ANGSD program. The 15 samples *Atalantia buxifolia* samples were used as the outgroup, while the 11 samples from the WILDSEX1 group were used as the sister clade. The samples from WILDAPO and WILDSEX2 were distinguished with different color. The two groups of CULAPO and CULSEX were combined as the CUL group.
(TIF)

S17 Fig The network phylogeny of wild and cultivated groups was performed based on the species-specific variations. The sequences were generated from the species-specific variations, and the network phylogeny was constructed using the SplitTree program with the default parameters.
(TIF)

S18 Fig The genome-wide *f*_*d*_ statistics are calculated in two combinations to investigate the introgression from cultivated populations. The statistics were performed using the population-level variation map based on two combinations (WILDSEX1, WILDAPO; CUL, Outgroup) and (WILDSEX1, WILDSEX2; CUL, Outgroup). The two groups of CULAPO and CULSEX were combined as the CUL group.
(TIF)

S19 Fig Evaluation of the introgression from cultivated population into wild population using species-specific markers (SSMs). The heatmap shows heterozygous or homozygous introgressed fragments in the three samples from WILDAPO (apomictic verities FC01, SY02 and WH) and three samples from WILDSEX2 (sexually reproducing verities NXG-2, DSD-2 and LZSZ-1). The x-axis indicated the genome reference of nine chromosomes.
(TIF)

S20 Fig The unfolded site frequency spectrum (uSFS) of synonymous mutations (sSNPs) and deleterious mutations (dSNPs) in three wild groups. The sSNPs and dSNPs are calculated based on genome-wide variation data set. (A) The uSFS was performed using the variations in 24 wild apomictic samples from the WILDAPO group. (B) The uSFS was performed using the variations in 11 wild sexually reproducing samples from the WILDSEX1 group. (C) The uSFS was performed using the variations in 17 wild sexually reproducing samples from the WILDSEX2 group.
(TIF)

S21 Fig The number of heterozygous deleterious alleles in five groups of Fortunella. The samples from different groups are distinguished with different color. The x-axis indicated the individuals ordered based on the numbers.
(TIF)

S22 Fig The diagram of deleterious alleles under the different reproductive patterns. There are three potential reproductive patterns in the apomictic wild kumquat. The leaky sexual reproductions were highlighted with dotted lines (Selfing and Outcrossing). Apomixis could keep the heterozygous deleterious alleles in heterozygous state. The outcrossing might keep the heterozygous deleterious alleles in heterozygous state when hybrid with unrelated individuals, whereas the selfing will lead to the deleterious alleles in homozygous state in the next generation. This image is made by the author and under CC BY 4.0 license.
(TIF)

S23 Fig The standardized haplotype distance (divergence) in each sample from five groups. The distance of two haplotypes within each sample was calculated based on a 25 kb non-overlap window. The standardized haplotype distance was similar with the pattern of heterozygosity in five groups, supporting the reliability of our analysis. **, *P* < 0.01 (Student t-test).
(TIF)

S24 Fig The divergence between two haplotypes in each apomictic sample and the distance to haplotypes from the WILDSEX1 group. The distance of two haplotypes within the samples from the WILDAPO group and the distance to haplotypes from the WILDSEX1 group were calculated based on a 25 kb non-overlap window. The Hap1 and Hap2 indicated the two haplotypes from the sample in the WILDAPO group, while the Hap2 represented the haplotype that did not clustered with the WILDAPO group. The Hap1-Hap2 indicated the standardized haplotype distance of two haplotypes. The Hap2-WILDSEX1 indicated the standardized haplotype distance of Hap2 and the haplotypes from the WILDSEX1 group.
(TIF)

S25 Fig The model of forward simulation to evaluate the influence on genetic diversity of putative population with different reproductive systems. The demographic model used in forward simulation was inferred from the WILDSEX1 group using the SMC++program. Going forward in time, after a burn-in period of 10*N generations (100k generations), the ancestral sexual population splits into two subpopulations with 0.1*N population size, one capable of selfing (selfing rate = 1), one capable of outcrossing (selfing rate = 0) The dashed lines represent the time of demographic shift reproductive types. The parameters were simulated independently with 100 repeats.
(TIF)

S26 Fig The Integrative Genomics Viewer (IGV) of two S-alleles based on the reads mapping. There were two S-alleles in the sexually reproducing sample ‘QLS’ from the WILDSEX2 group. (A) The IGV plot indicated the coverage of short-paired reads in the S9 sequences. The directions of reads were indicated by different colors. (B) The IGV plot indicated the coverage of short-paired reads in the S23 sequences. The directions of reads were indicated by different colors.
(TIF)

S1 Table Geographical occurrences of 396 *Fortunella* samples. (XLSX)

S2 Table Standardized 19 climatic variables of 396 *Fortunella* samples. (XLSX)

S3 Table Statistics of genome sequence of *Fortunella* and outgroup accessions used in this study. (XLSX)

S4 Table *ABBA-BABA* statistics detected introgression in combinations of five groups. (XLSX)

S5 Table Estimation of introgression in samples from WILDAPO and WILDSEX2 groups using species-specific variations. (XLSX)

S6 Table Heterozygosity for each accession based on the variation map. (XLSX)

S7 Table Statistics of *S*-locus genotypes in nine samples from the WILDSEX1 group and four samples from the WILDSEX2 group. (XLSX)
