# Supplementary figures and images for: Genomic conservation of crop wild relatives: A case study of citrus
Source: PLoS Genet. 2023 Jun 20;19(6):e1010811. doi: 10.1371/journal.pgen.1010811 (PMC10321653; doi:10.1371/journal.pgen.1010811)

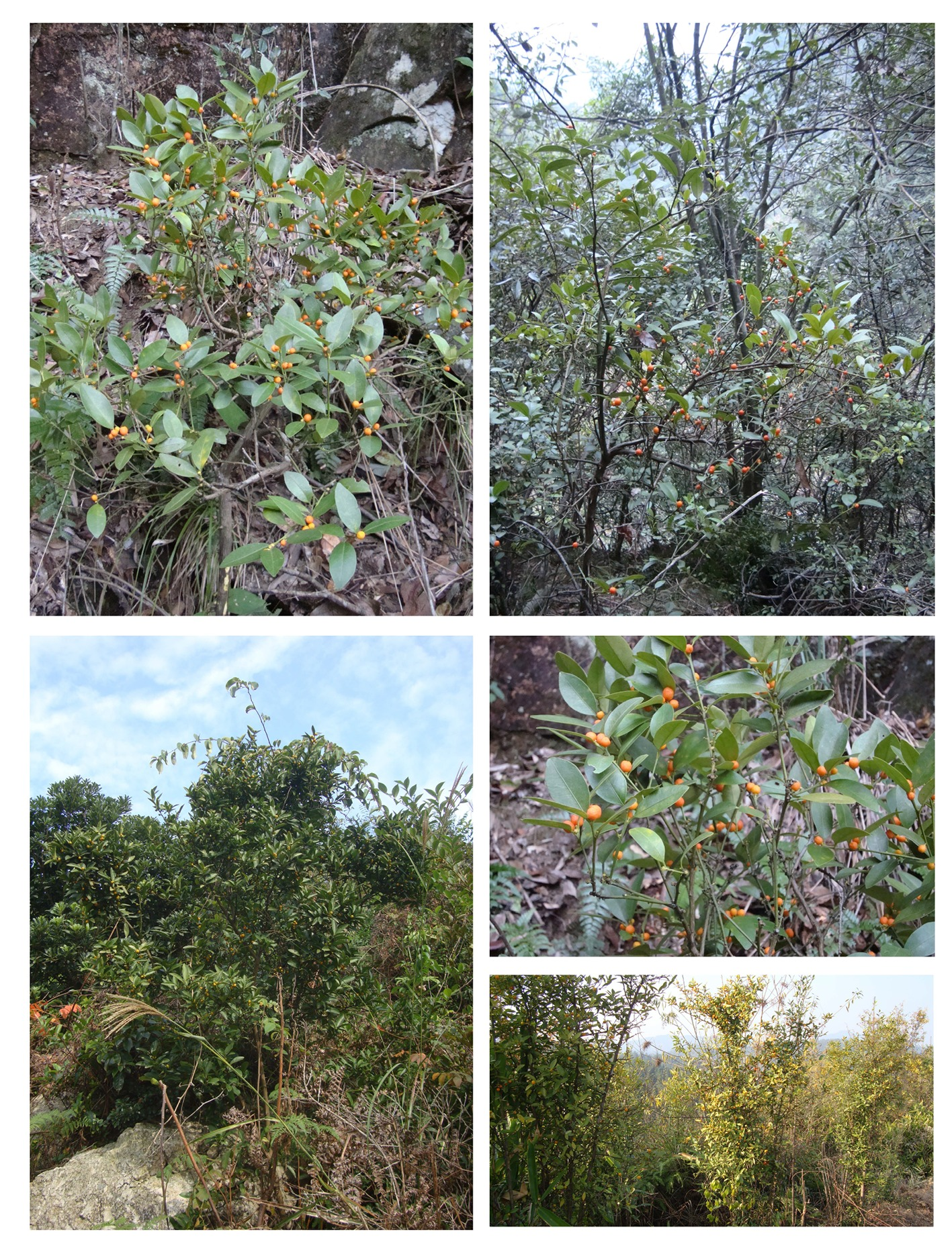

Supplement: S1 Fig — The wild kumquats are mostly grown on hillsides, with low distribution density. (TIF) [file pgen.1010811.s001.tif]

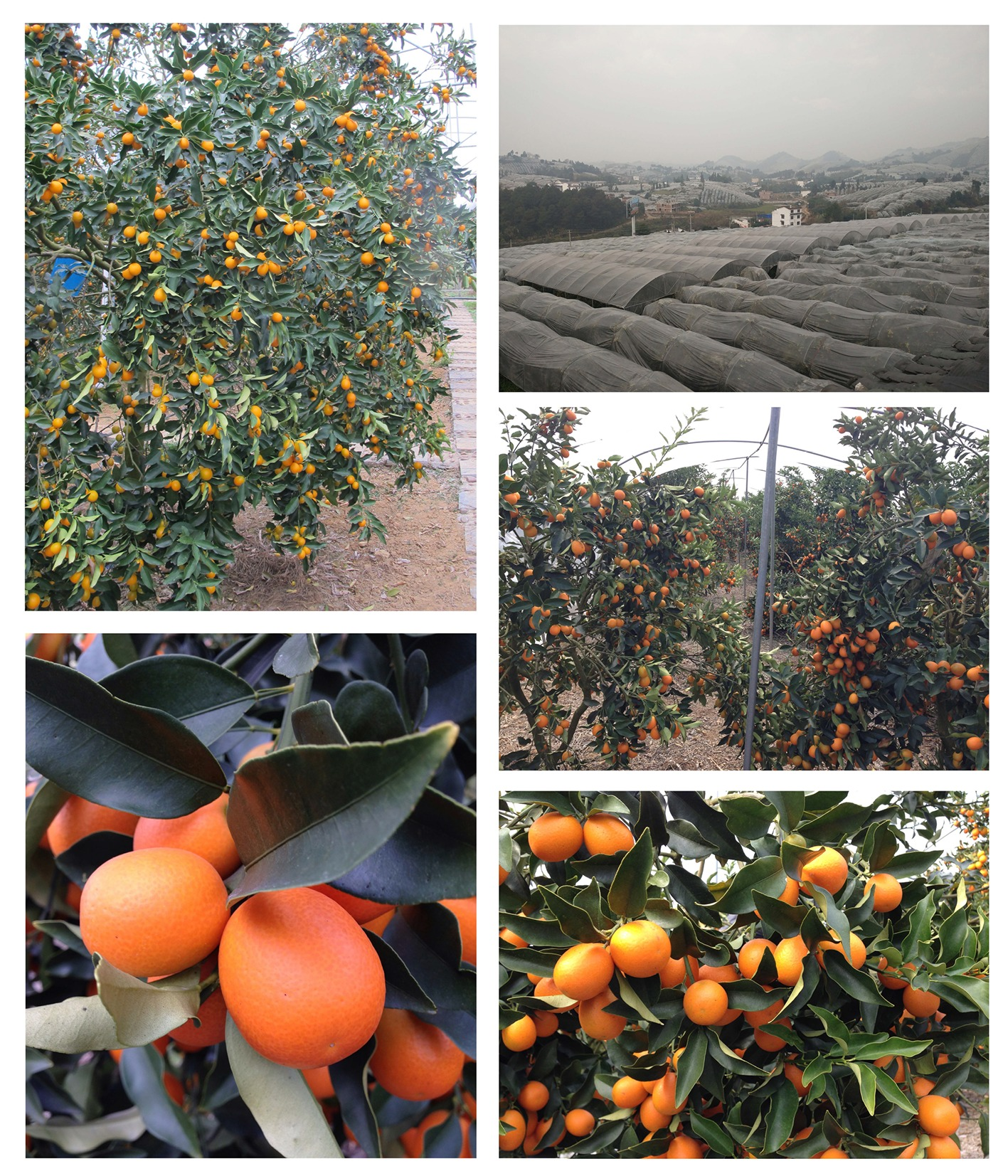

Supplement: S2 Fig — The cultivated kumquat is a kind of intensive farming with uniform genotype by grafting. There are a series of agricultural technologies in this process. (TIF) [file pgen.1010811.s002.tif]

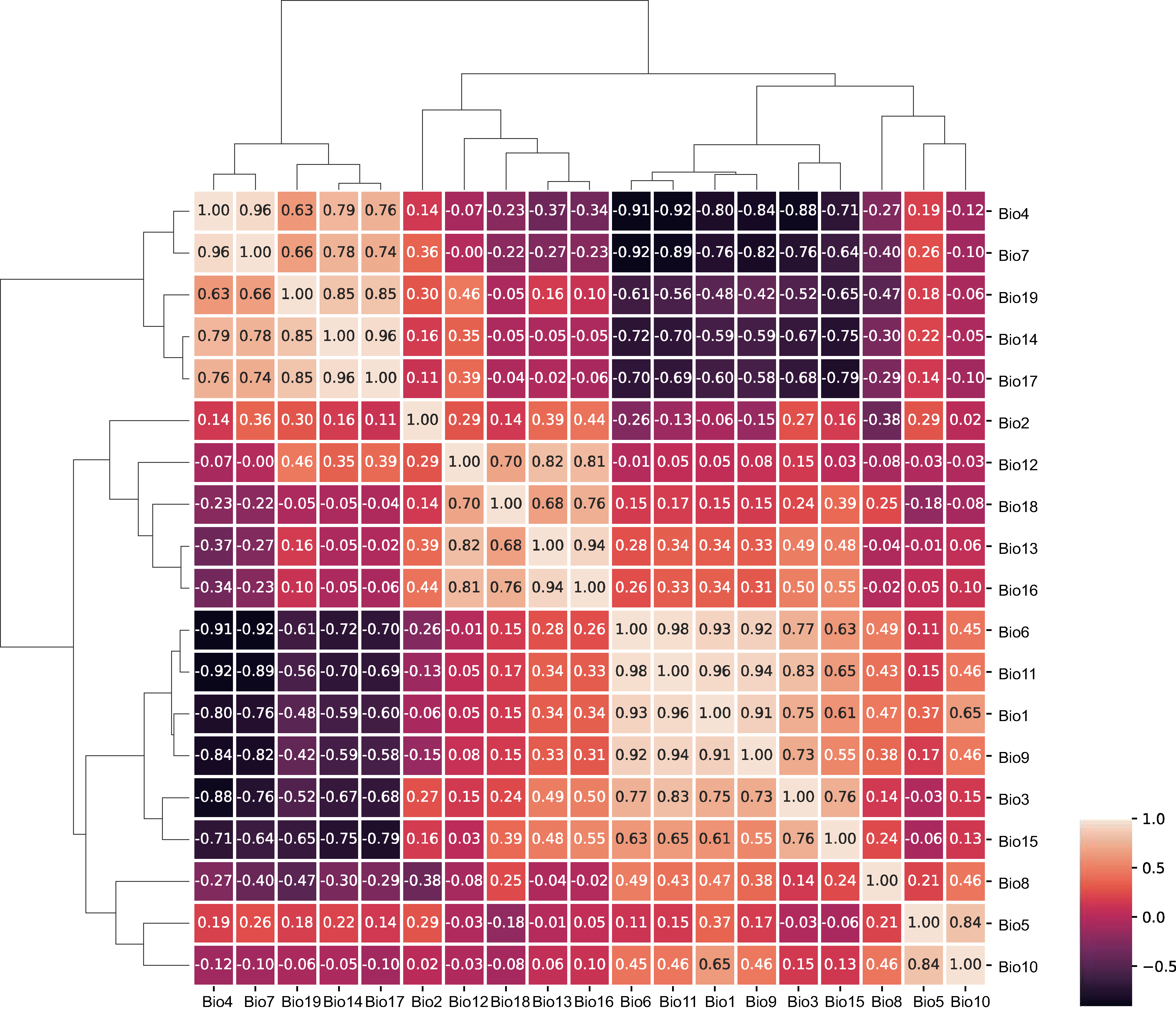

Supplement: S3 Fig — Bioclimatic variables were derived from the monthly temperature and rainfall values to generate more biologically meaningful variables. The bioclimatic variables represented annual trends, seasonality and extreme or limiting environmental factors. The bioclimatic matrix was generated based on the 396 geographical data of Fortunella genus. (TIF) [file pgen.1010811.s003.tif]

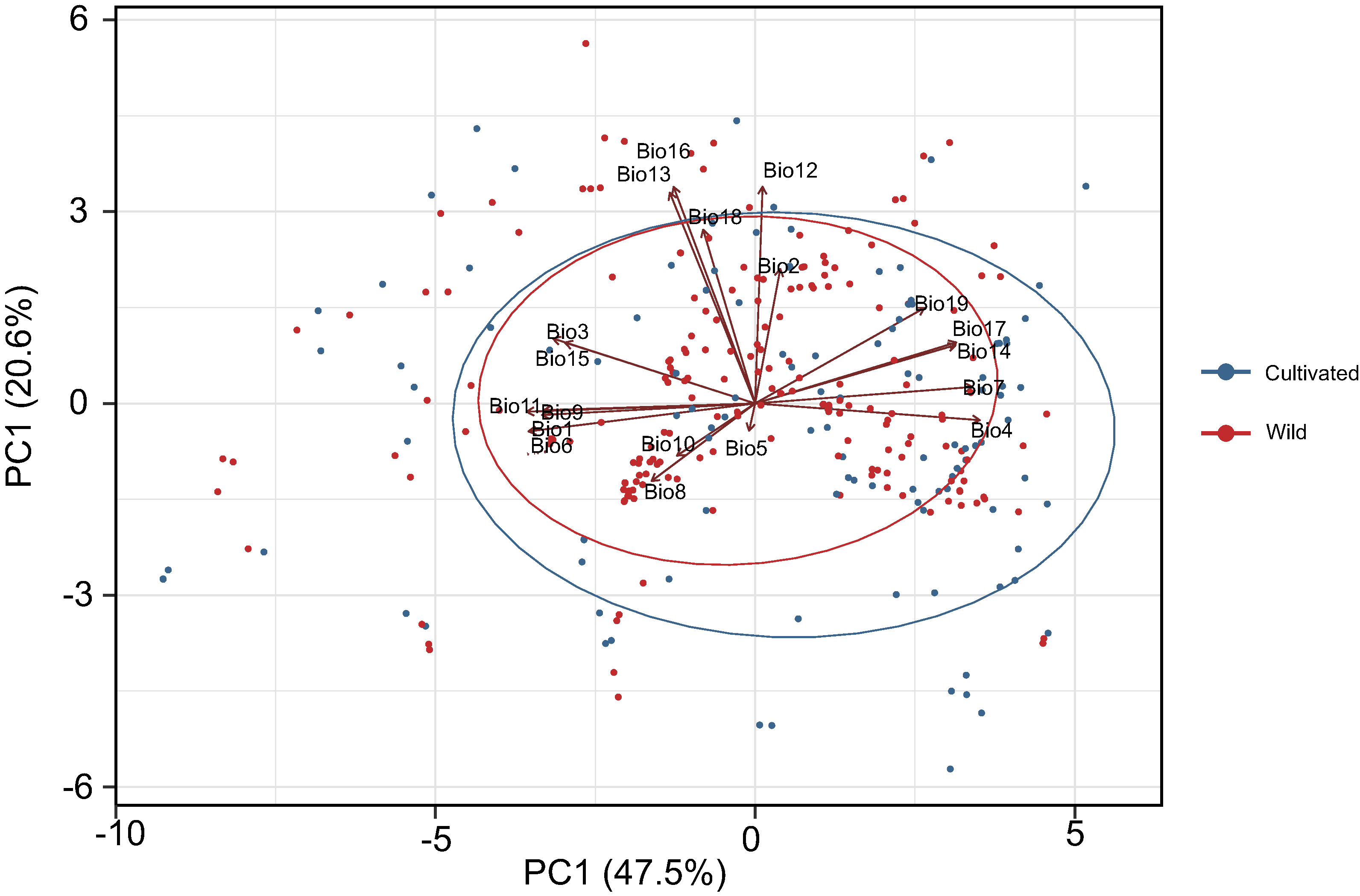

Supplement: S4 Fig — The PCA analysis was constructed based on bioclimatic matrix of the 396 geographical data and used to investigate the most informative variables related to the distribution of wild and cultivated samples. PC1 represented the first principal component with interpretation of 47.5% variations. PC2 represented the second principal component with interpretation of 20.6% variations. The wild and cultivated populations were distinguished with different color. Each characteristic influences a principal component is highlighted. (TIF) [file pgen.1010811.s004.tif]

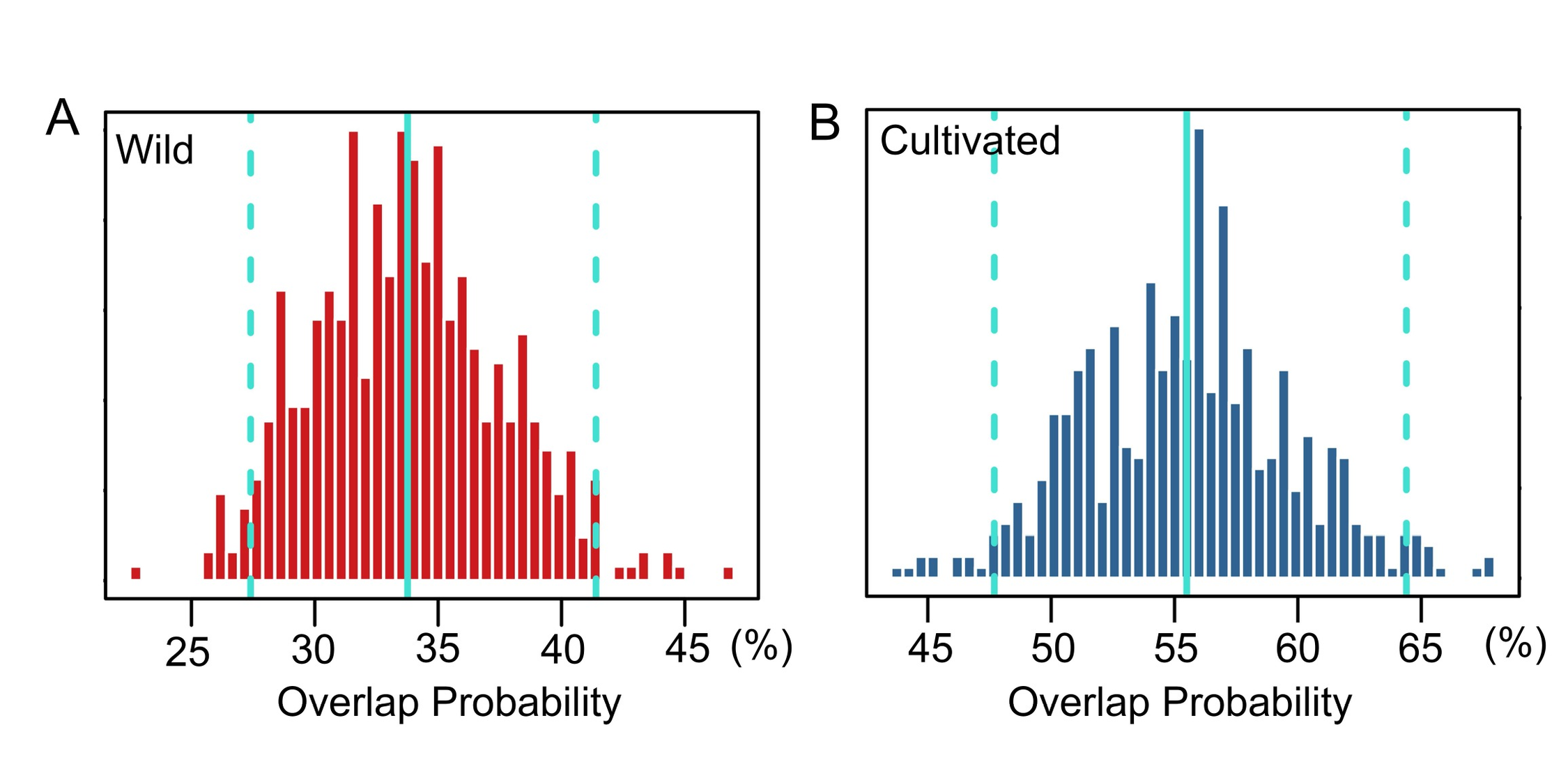

Supplement: S5 Fig — Niche overlap was calculated as the probability that an individual from species A is found in the niche region of species B. The niche regions and pairwise niche overlap of wild and cultivated populations in Fortunella were calculated using 19 environmental variables. (A) The distribution of overlap probability that an individual from cultivated population was found in the niche region of the wild population. (B) The distribution of overlap probability that an individual from wild population was found in the niche region of domesticated population. The solid line showed the average of niche overlap probability and the dashed lines showed 95% probability of niche overlap probability. (TIF) [file pgen.1010811.s005.tif]

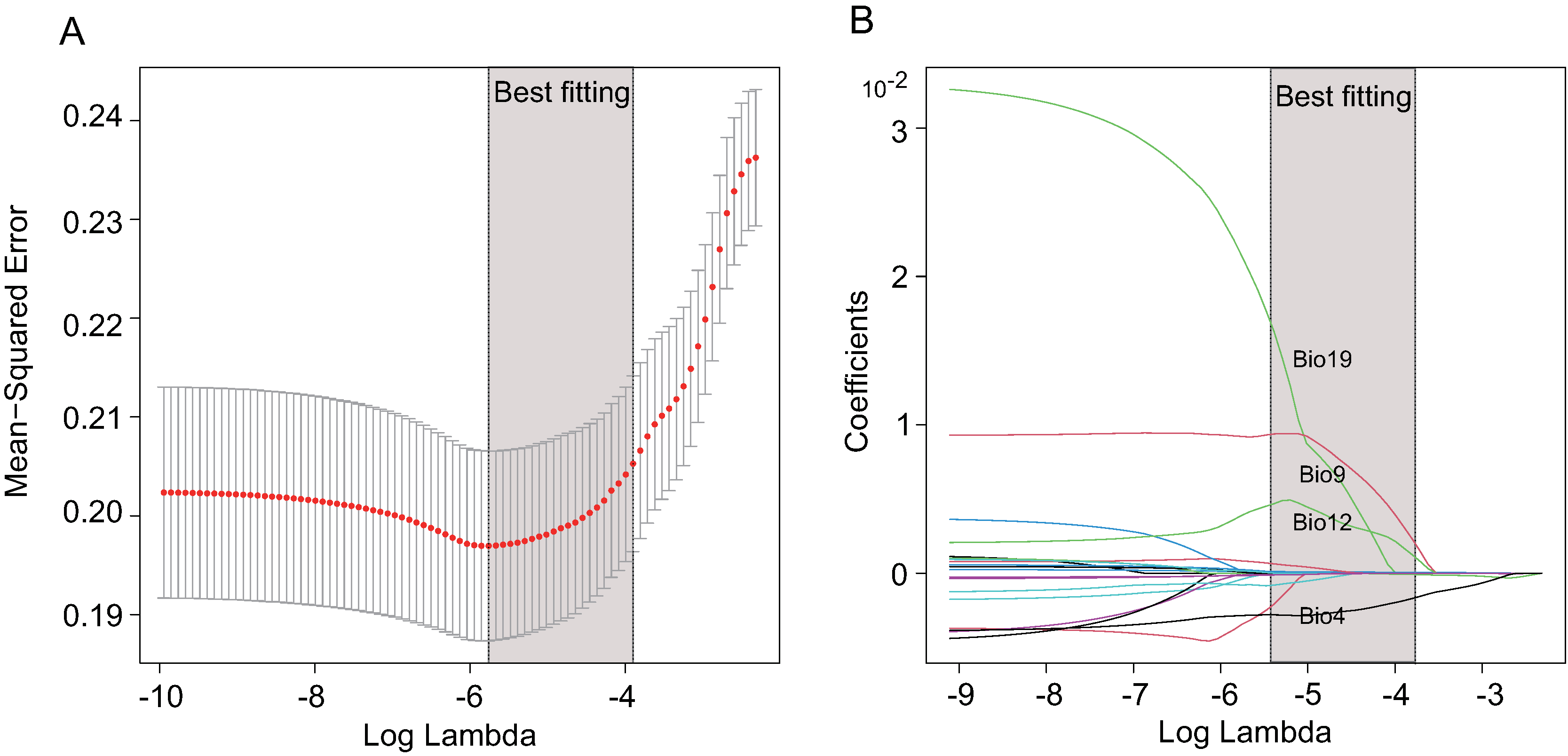

Supplement: S6 Fig — The bioclimatic matrix of Fortunella genus was used to predict using the Glmnet program. (A) The cross-validated fit for selected values of lambda (log scale). (B) The coefficients curve corresponds to variables were plotted. The best fitting was highlighted. The y-axis indicated the number of coefficients at the current lambda. (TIF) [file pgen.1010811.s006.tif]

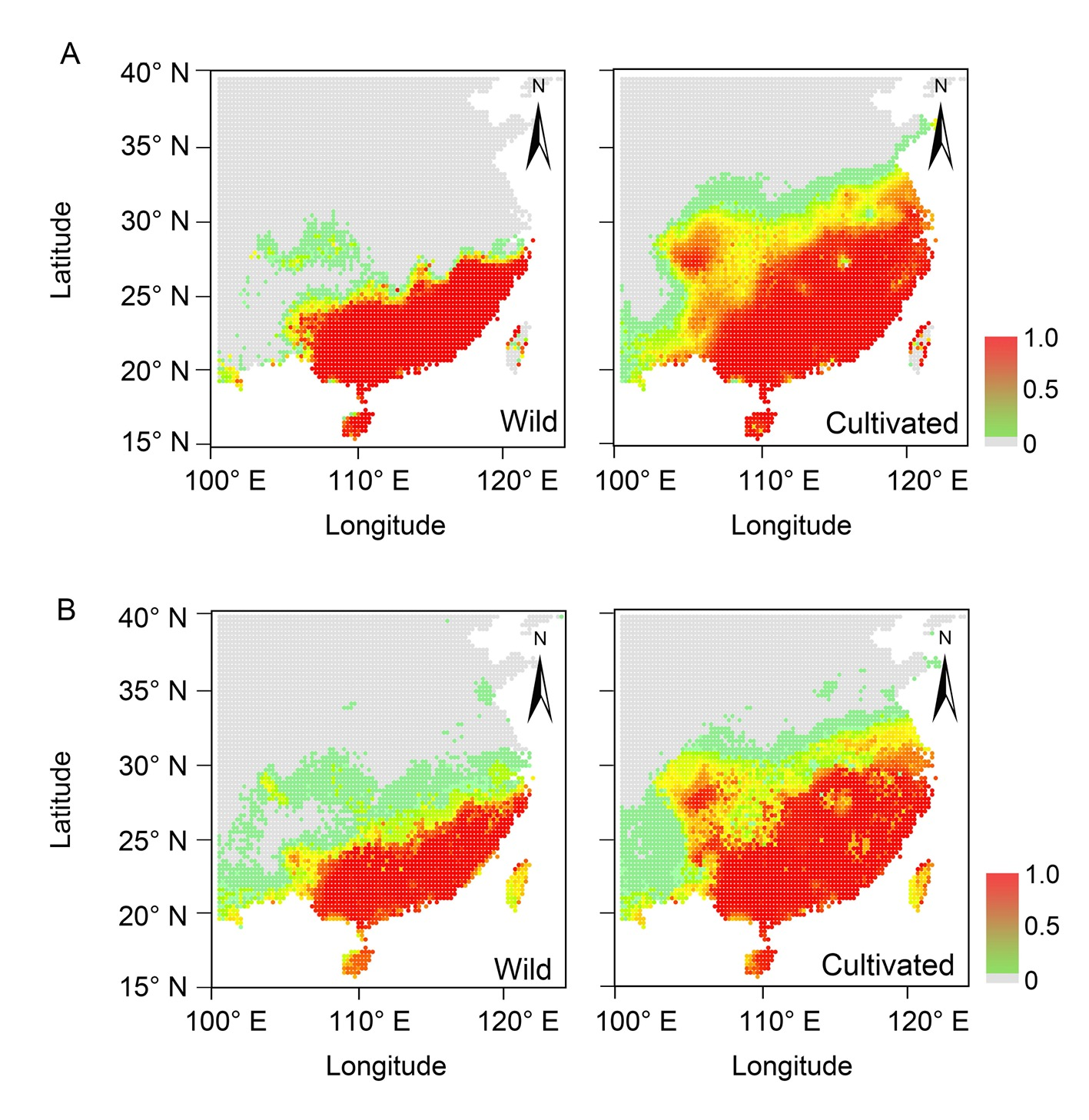

Supplement: S7 Fig — The 19 bioclimatic variables were used for distribution modeling. The heatmap showed the predicted distribution of wild and domesticated populations using Generally Linear Model (GLM) and Generalized Boosting Model (GBM) models, respectively. The color presented the probability of prediction, and the resolution is 5 min. The base layer of the map from Tianditu, the National Platform for Common Geospatial Information Services (NPCGIS) https://www.tianditu.gov.cn/. The base layer is under CC BY 4.0 license. (TIF) [file pgen.1010811.s007.tif]

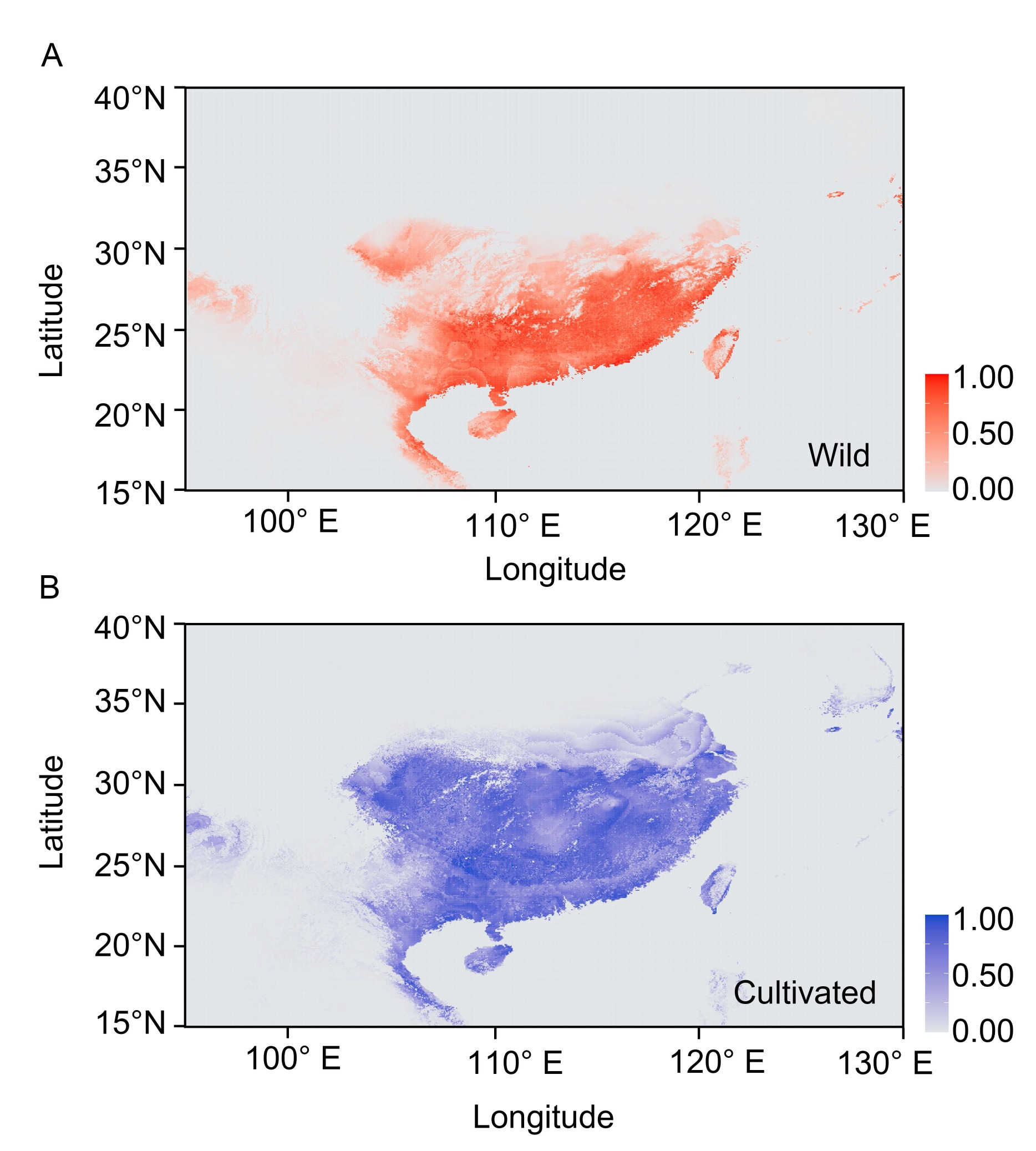

Supplement: S8 Fig — The heatmap showed the predicted distribution with default parameters and generated with 100 iterations. (A) The predicted distribution of wild kumquats. (B) The predicted distribution of cultivated kumquats. The base layer of the map from Tianditu, the National Platform for Common Geospatial Information Services (NPCGIS) https://www.tianditu.gov.cn/. The base layer is under CC BY 4.0 license. (TIF) [file pgen.1010811.s008.tif]

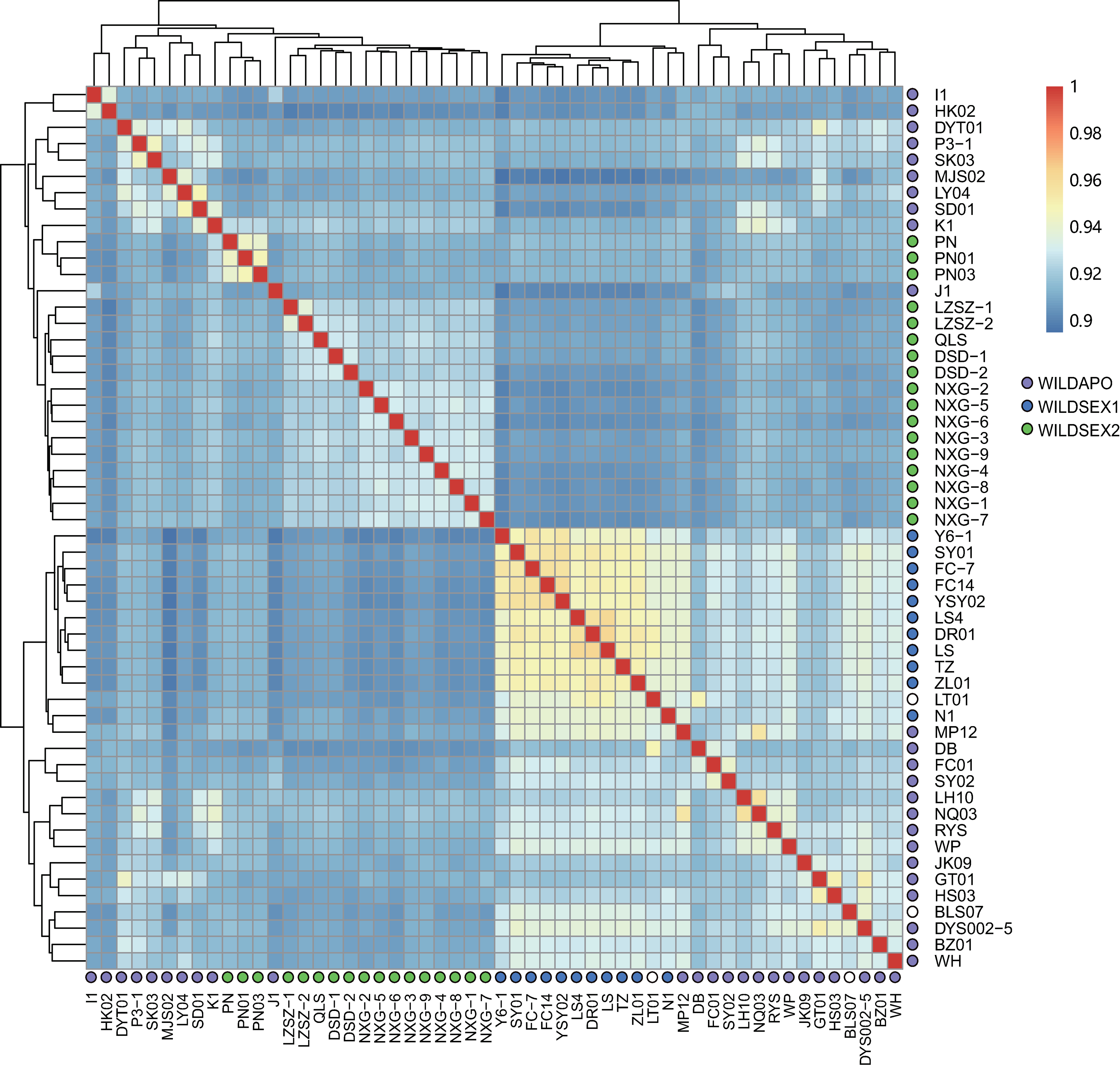

Supplement: S9 Fig — The pairwise IBD values were calculated based on genome-wide variations of the 54 sequenced samples in Fortunella using PLINK. The samples from different groups were distinguished with different color. (TIF) [file pgen.1010811.s009.tif]

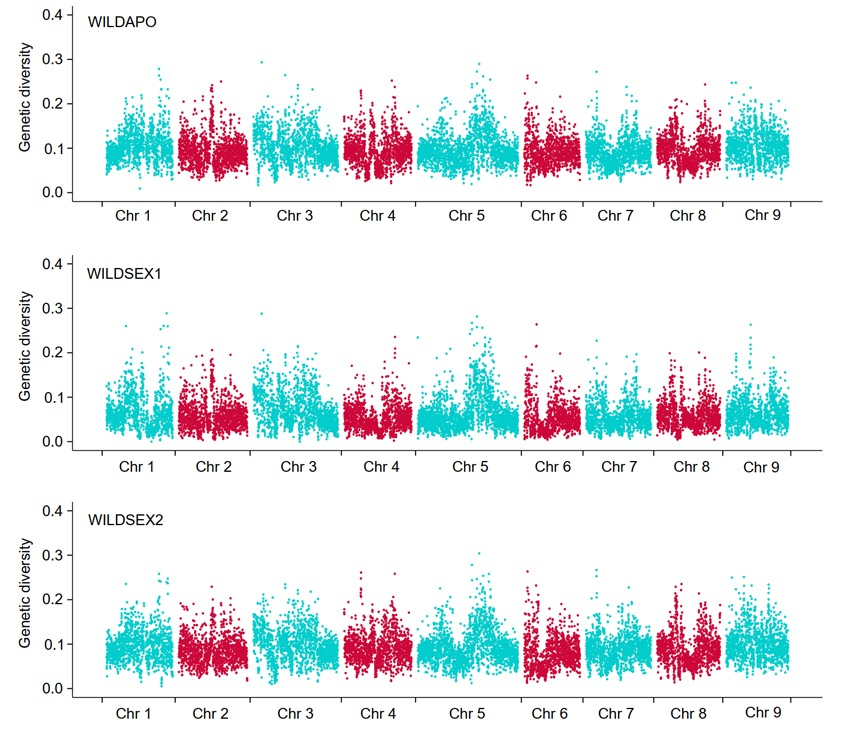

Supplement: S10 Fig — The genome-wide genetic diversity (π) of the WILDAPO, WILDSEX1 and WILDSEX2 groups were calculated based on the variation map. Those statistics were calculated based on 25 kb non-overlapping windows. (TIF) [file pgen.1010811.s010.tif]

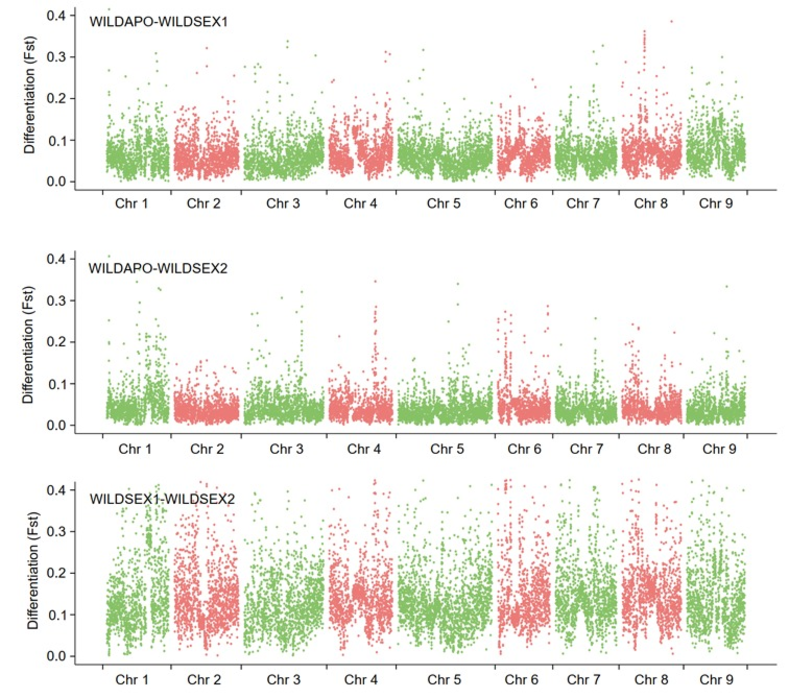

Supplement: S11 Fig — The genome-wide differentiation (Fst) was performed between three combinations: between WILDAPO and WILDSEX1 groups, between WILDAPO and WILDSEX2 groups, and between WILDSEX1 and WILDSEX2 groups. Those statistics were calculated based on 25 kb non-overlapping windows. (TIF) [file pgen.1010811.s011.tif]

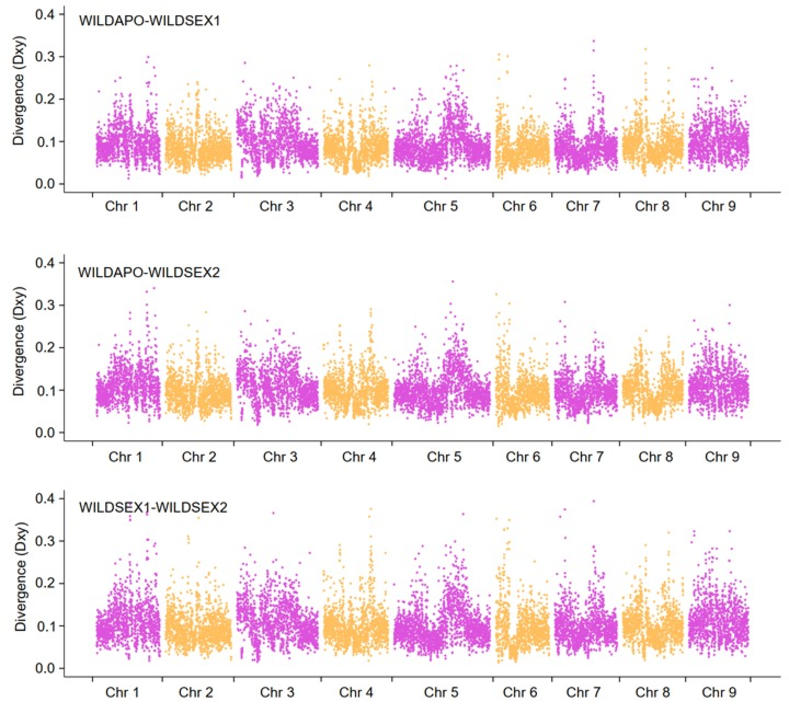

Supplement: S12 Fig — The genome-wide divergence (Dxy) was performed between three combinations: between WILDAPO and WILDSEX1 groups, between WILDAPO and WILDSEX2 groups, and between WILDSEX1 and WILDSEX2 groups. Those statistics were calculated based on 25 kb non-overlapping windows. (TIF) [file pgen.1010811.s012.tif]

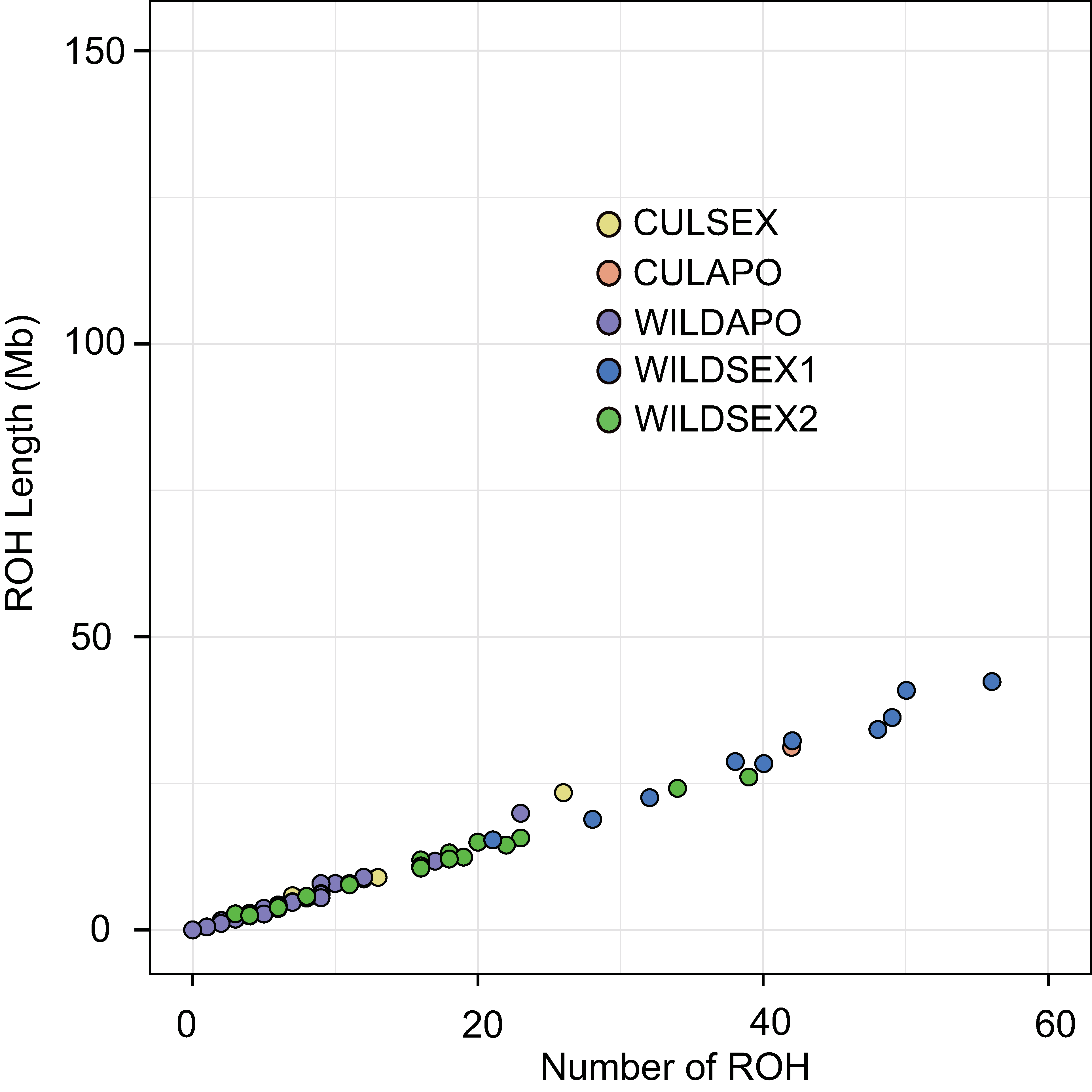

Supplement: S13 Fig — The x-axis indicated the number of ROHs, while the y-axis indicates the length of ROHs in the genome. The samples from different groups were distinguished with different color. (TIF) [file pgen.1010811.s013.tif]

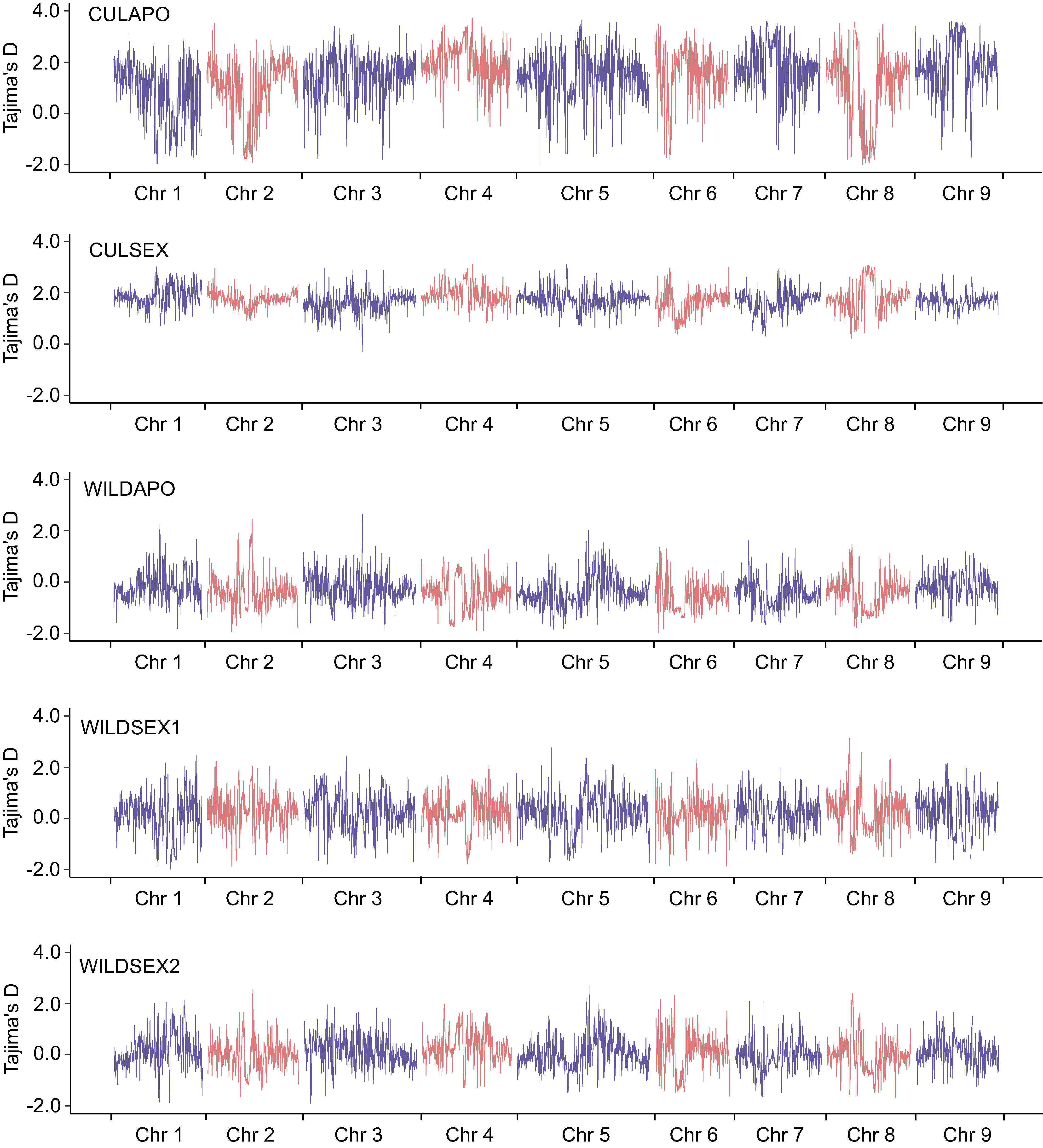

Supplement: S14 Fig — The genome-wide Tajima’s D of the CULAPO, CULSEX, WILDAPO, WILDSEX1 and WILDSEX2 groups are calculated based on the variation map. Those statistics were calculated based on 25 kb non-overlapping windows. (TIF) [file pgen.1010811.s014.tif]

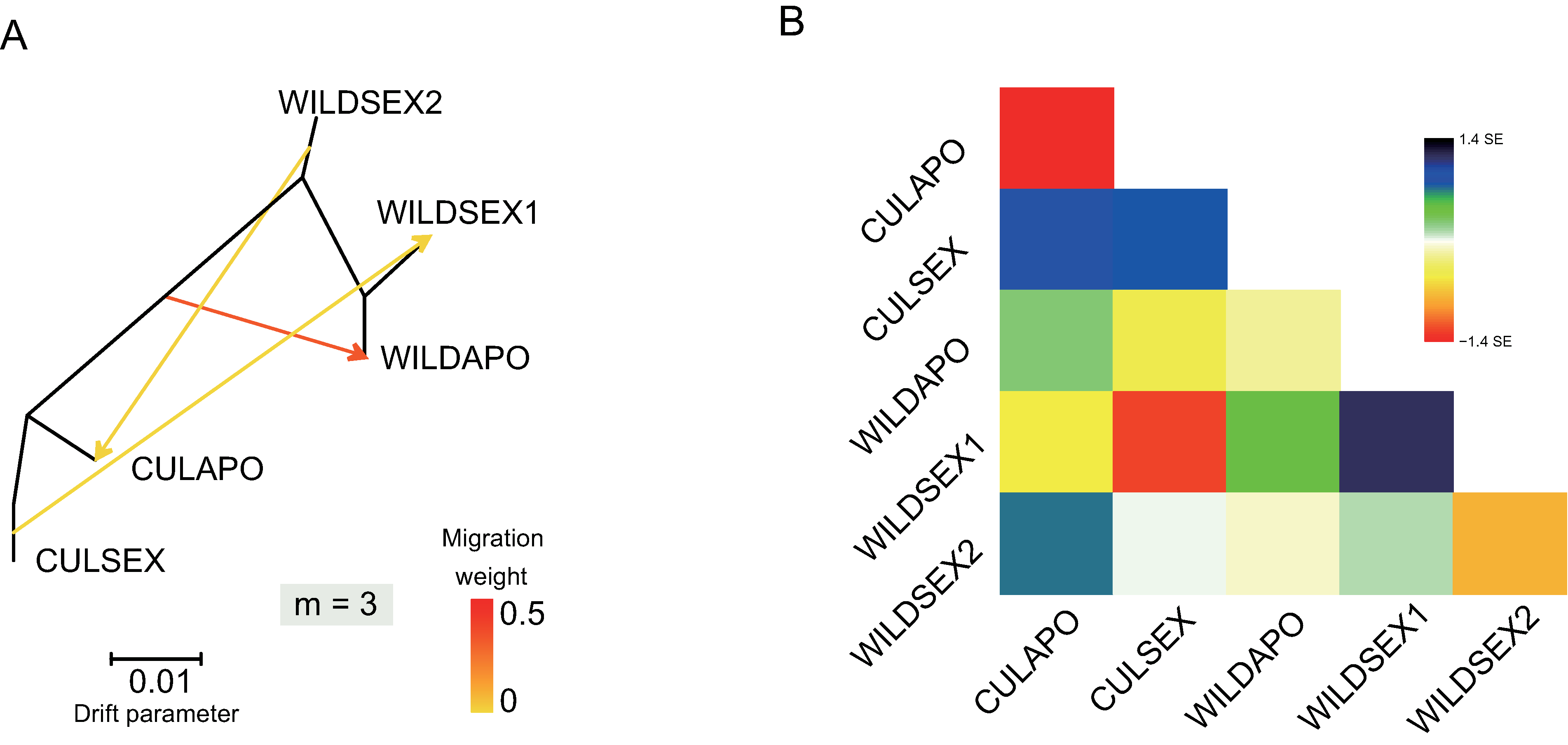

Supplement: S15 Fig — (A) The allele frequencies of five groups were used to estimate the graph structure and the potential introgression events with the parameter m = 3. (B) The pair wise residuals of the graph modeling were calculated based on the genome-wide allele frequencies in five groups. (TIF) [file pgen.1010811.s015.tif]

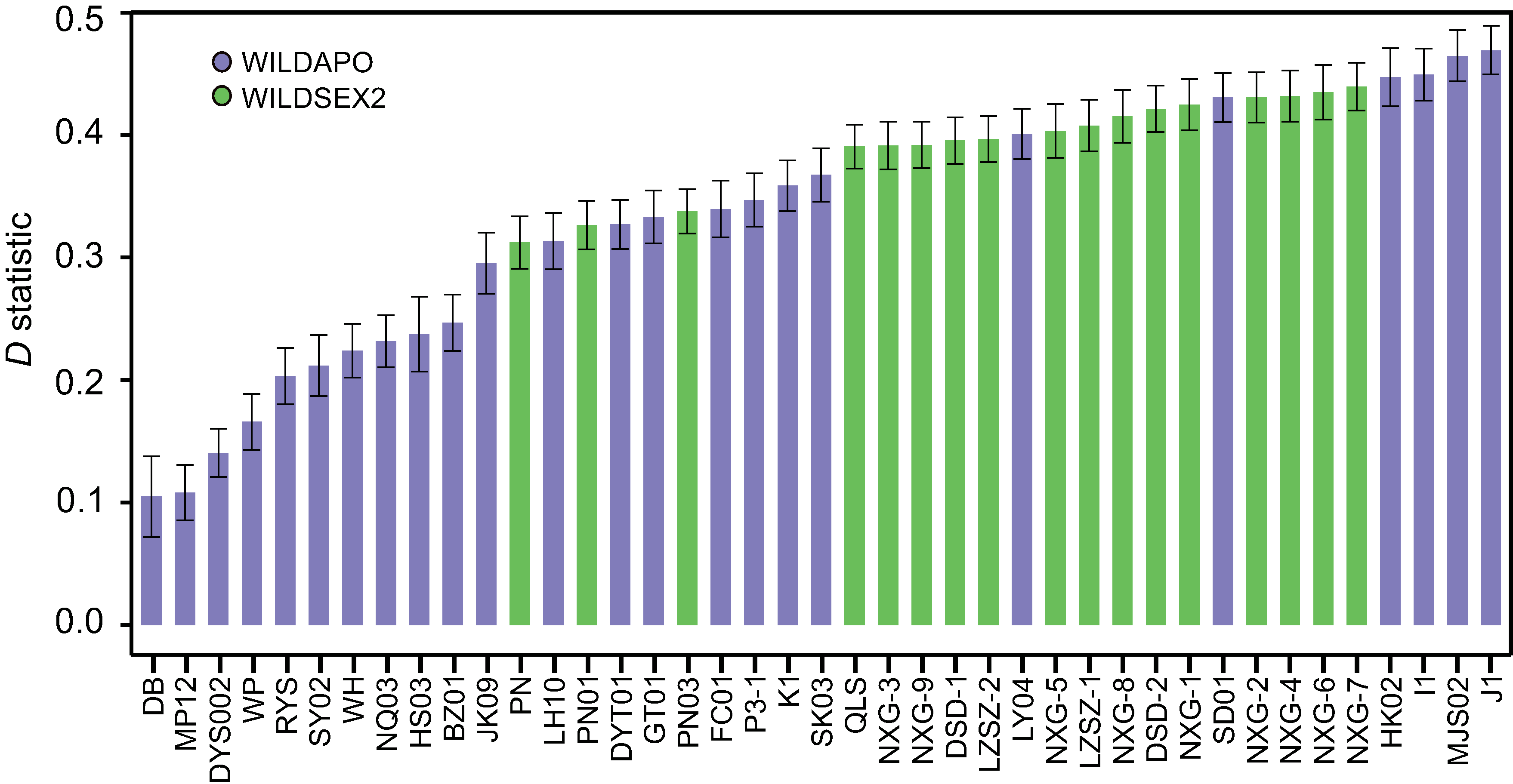

Supplement: S16 Fig — The D statistic was performed based on combinations of (WILDSEX1, WILDAPO; CUL, Outgroup) or (WILDSEX1, WILDSEX2; CUL, Outgroup) at individual-level using the ANGSD program. The 15 samples Atalantia buxifolia samples were used as the outgroup, while the 11 samples from the WILDSEX1 group were used as the sister clade. The samples from WILDAPO and WILDSEX2 were distinguished with different color. The two groups of CULAPO and CULSEX were combined as the CUL group. (TIF) [file pgen.1010811.s016.tif]

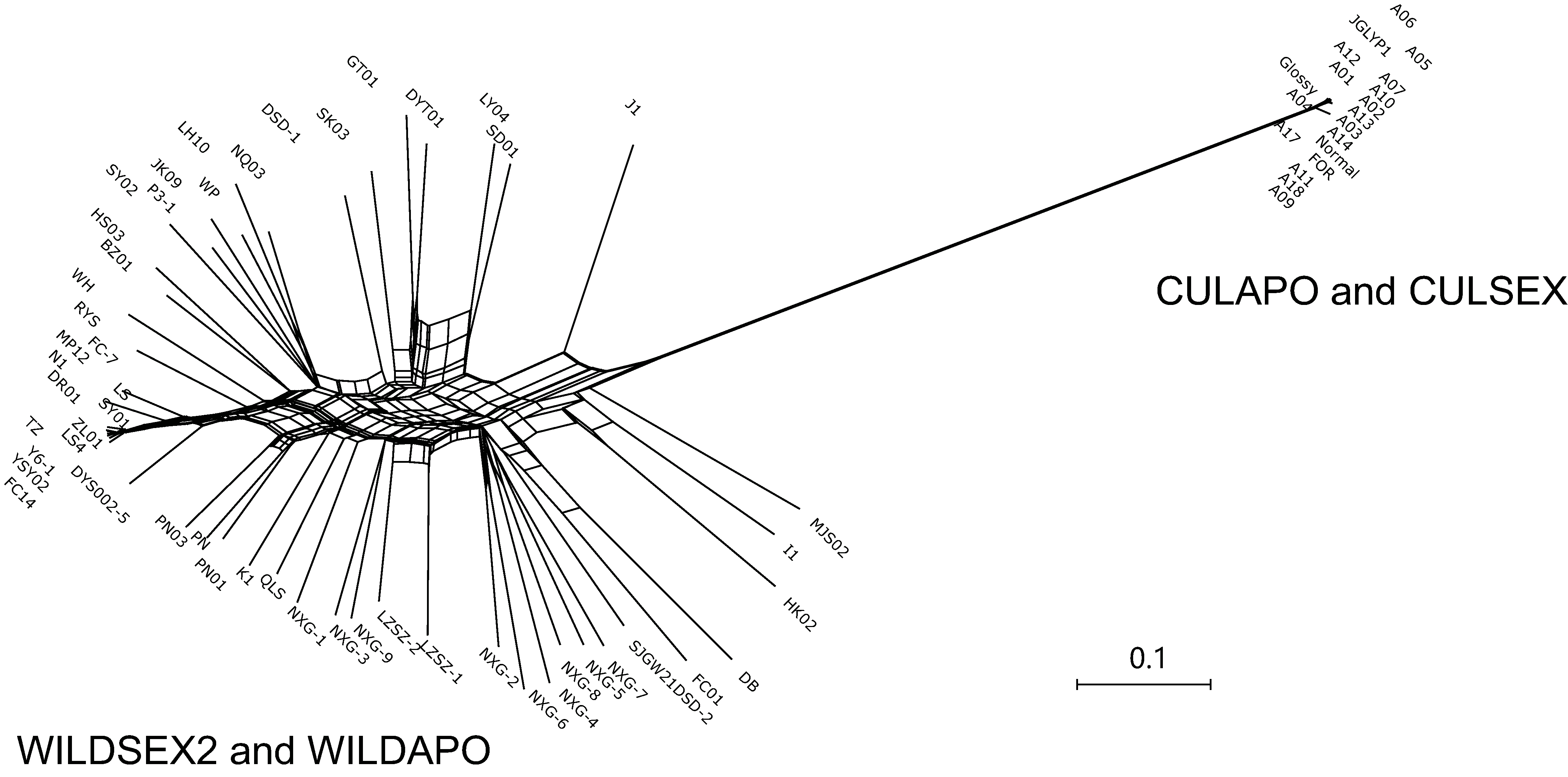

Supplement: S17 Fig — The sequences were generated from the species-specific variations, and the network phylogeny was constructed using the SplitTree program with the default parameters. (TIF) [file pgen.1010811.s017.tif]

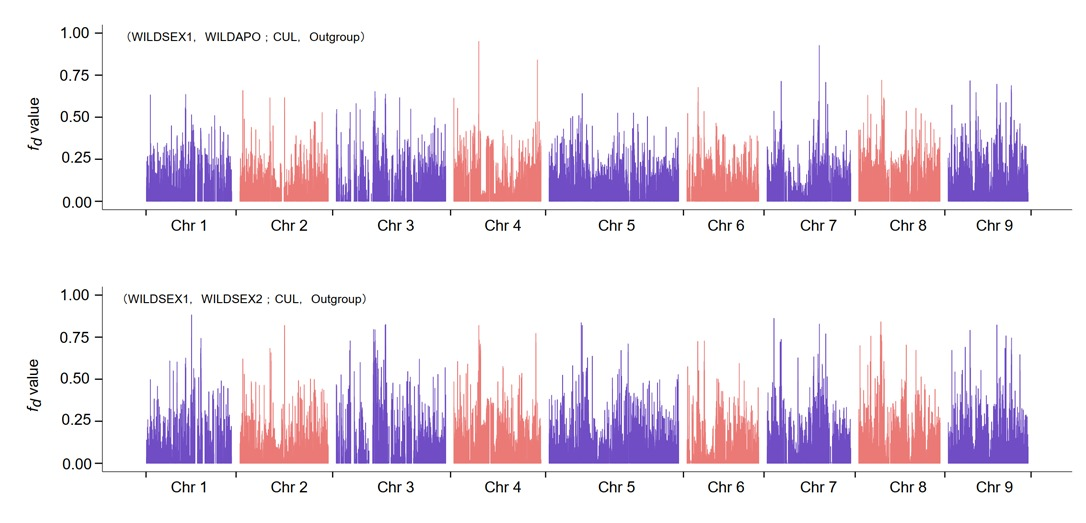

Supplement: S18 Fig — The statistics were performed using the population-level variation map based on two combinations (WILDSEX1, WILDAPO; CUL, Outgroup) and (WILDSEX1, WILDSEX2; CUL, Outgroup). The two groups of CULAPO and CULSEX were combined as the CUL group. (TIF) [file pgen.1010811.s018.tif]

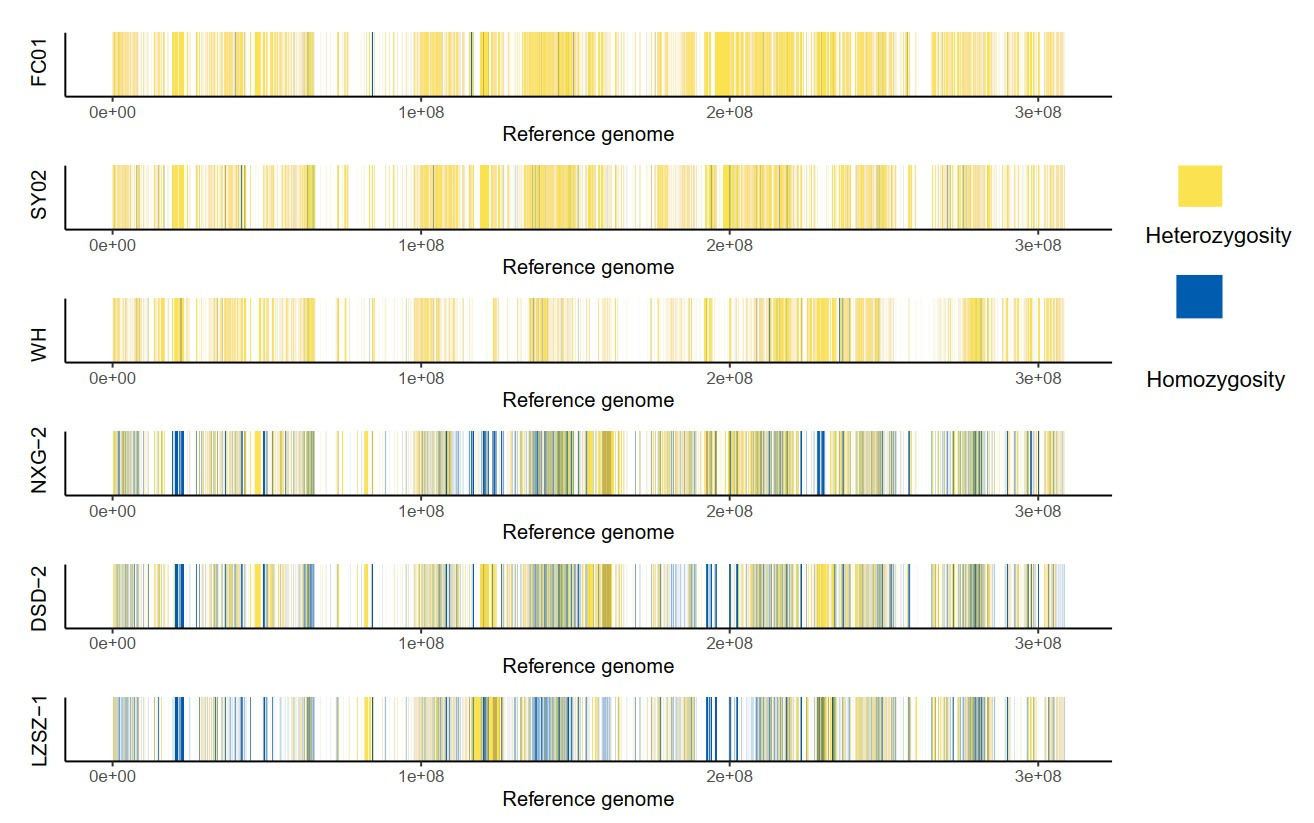

Supplement: S19 Fig — The heatmap shows heterozygous or homozygous introgressed fragments in the three samples from WILDAPO (apomictic verities FC01, SY02 and WH) and three samples from WILDSEX2 (sexually reproducing verities NXG-2, DSD-2 and LZSZ-1). The x-axis indicated the genome reference of nine chromosomes. (TIF) [file pgen.1010811.s019.tif]

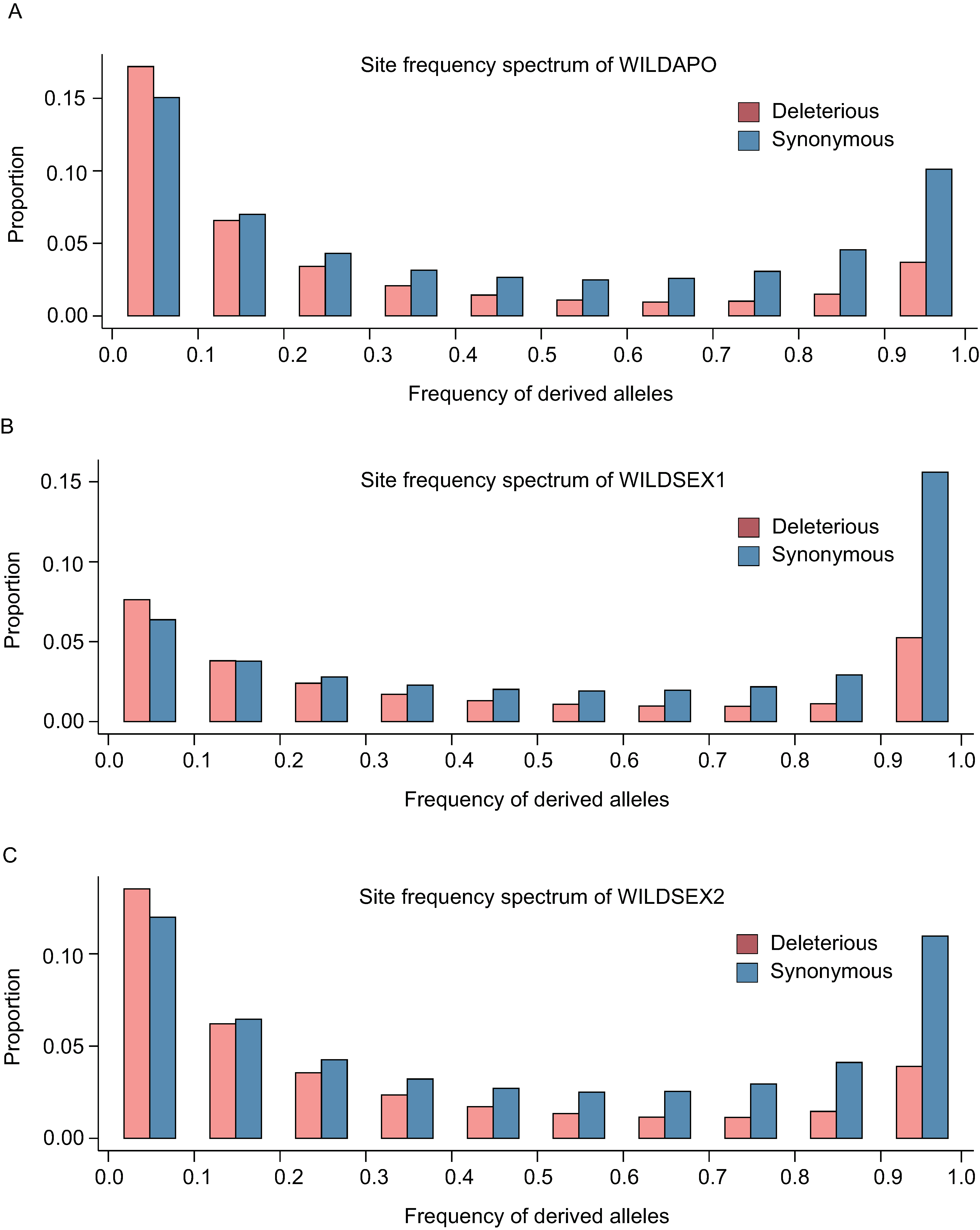

Supplement: S20 Fig — The sSNPs and dSNPs are calculated based on genome-wide variation data set. (A) The uSFS was performed using the variations in 24 wild apomictic samples from the WILDAPO group. (B) The uSFS was performed using the variations in 11 wild sexually reproducing samples from the WILDSEX1 group. (C) The uSFS was performed using the variations in 17 wild sexually reproducing samples from the WILDSEX2 group. (TIF) [file pgen.1010811.s020.tif]

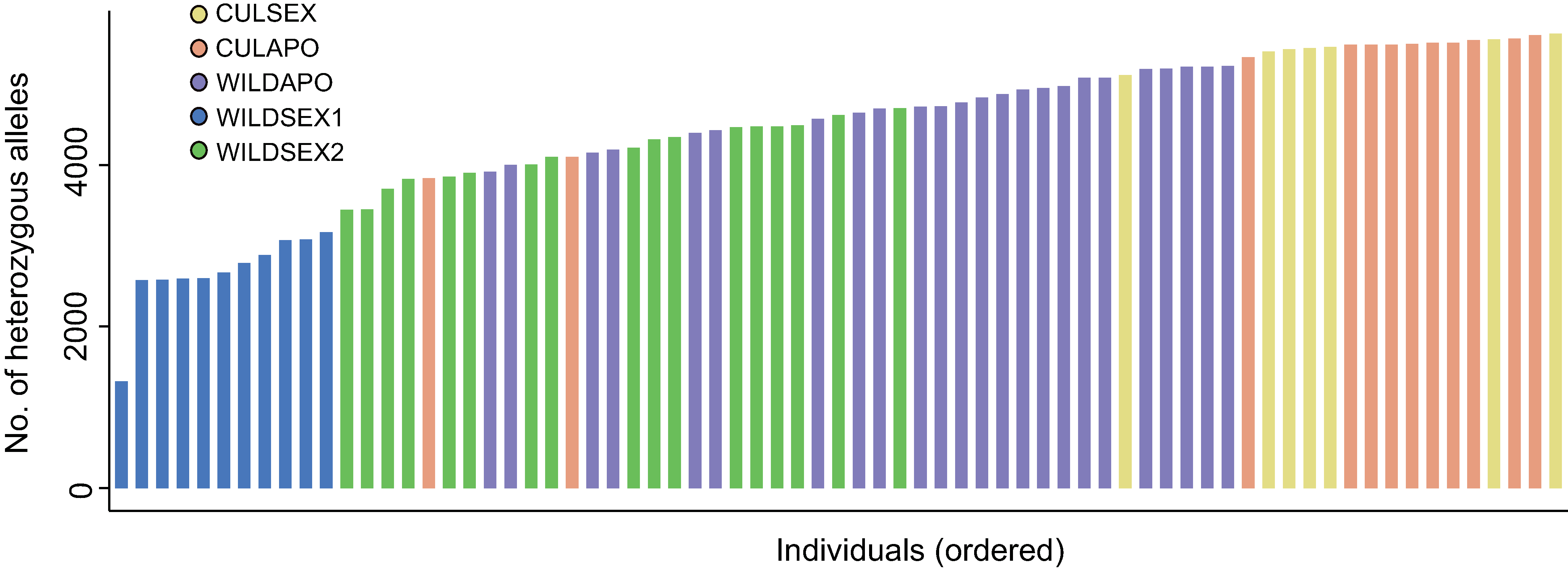

Supplement: S21 Fig — The samples from different groups are distinguished with different color. The x-axis indicated the individuals ordered based on the numbers. (TIF) [file pgen.1010811.s021.tif]

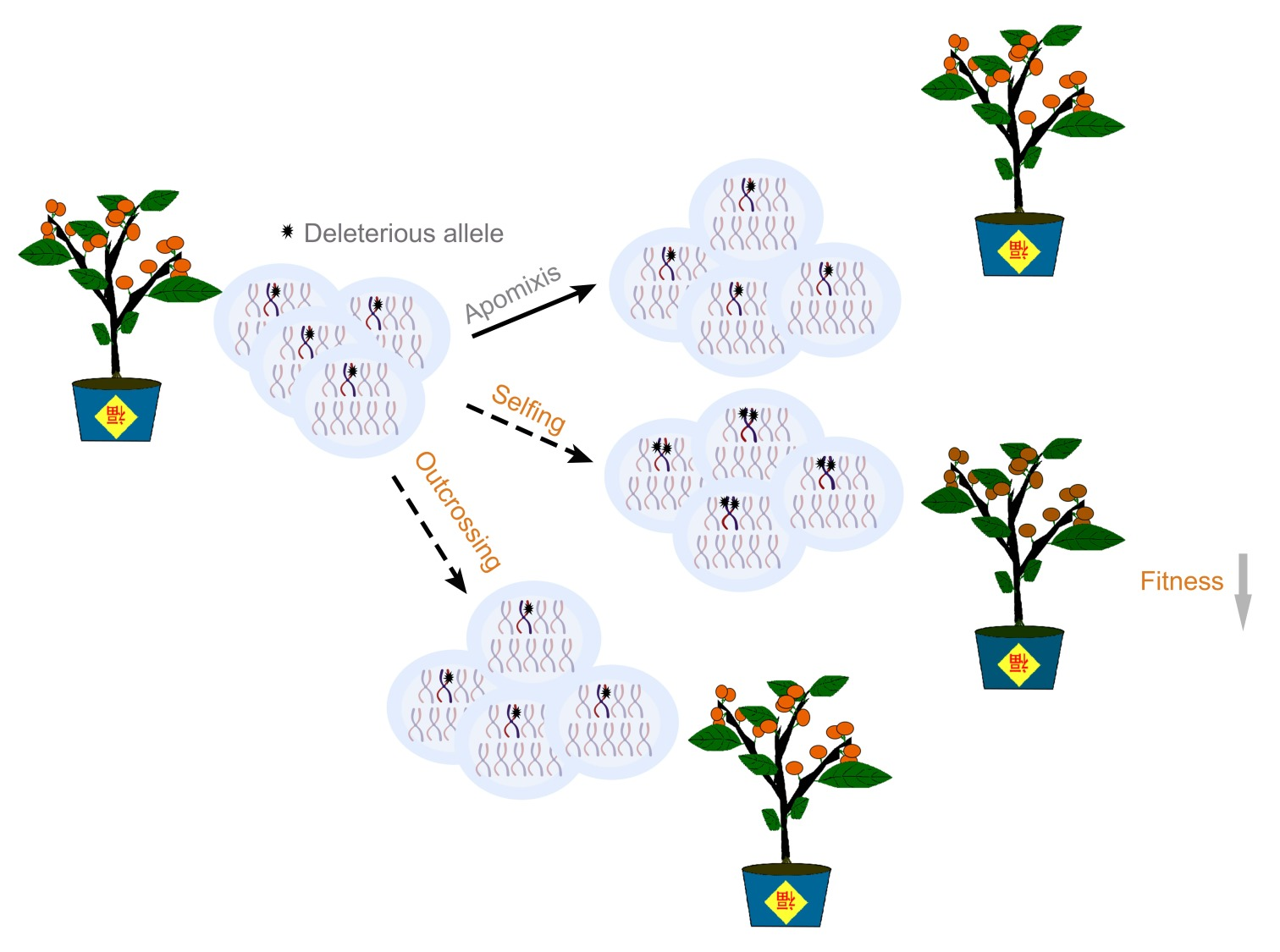

Supplement: S22 Fig — There are three potential reproductive patterns in the apomictic wild kumquat. The leaky sexual reproductions were highlighted with dotted lines (Selfing and Outcrossing). Apomixis could keep the heterozygous deleterious alleles in heterozygous state. The outcrossing might keep the heterozygous deleterious alleles in heterozygous state when hybrid with unrelated individuals, whereas the selfing will lead to the deleterious alleles in homozygous state in the next generation. This image is made by the author and under CC BY 4.0 license. (TIF) [file pgen.1010811.s022.tif]

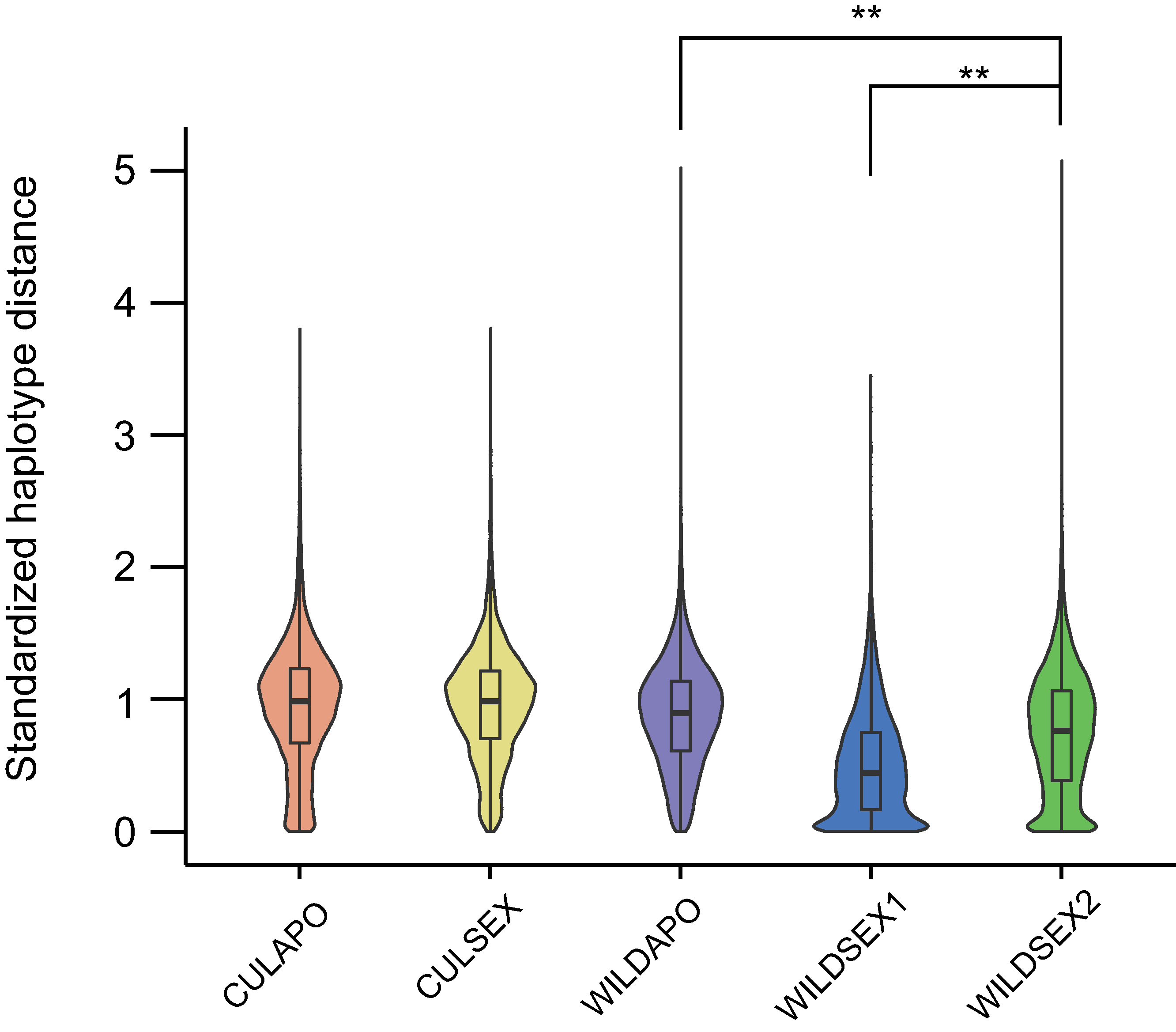

Supplement: S23 Fig — The distance of two haplotypes within each sample was calculated based on a 25 kb non-overlap window. The standardized haplotype distance was similar with the pattern of heterozygosity in five groups, supporting the reliability of our analysis. **, P < 0.01 (Student t-test). (TIF) [file pgen.1010811.s023.tif]

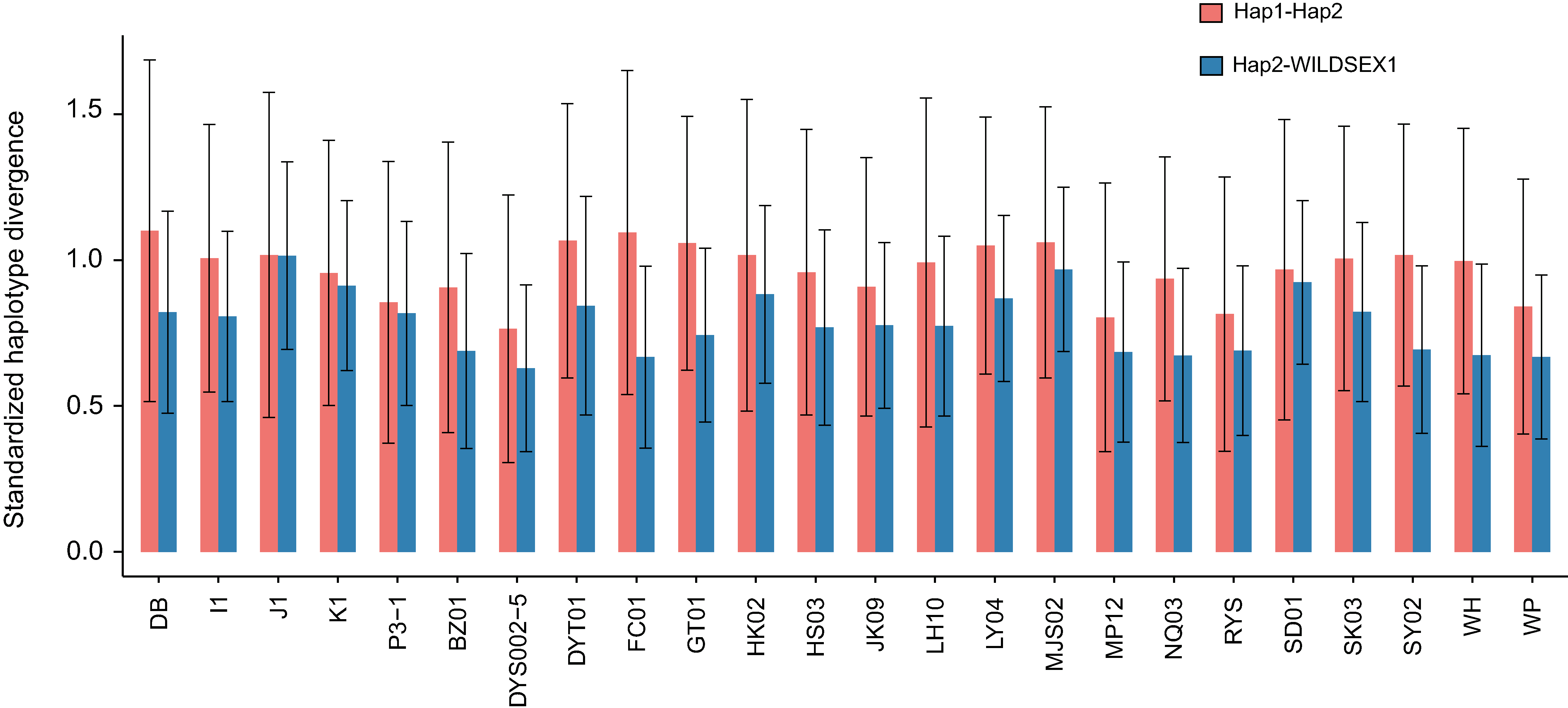

Supplement: S24 Fig — The distance of two haplotypes within the samples from the WILDAPO group and the distance to haplotypes from the WILDSEX1 group were calculated based on a 25 kb non-overlap window. The Hap1 and Hap2 indicated the two haplotypes from the sample in the WILDAPO group, while the Hap2 represented the haplotype that did not clustered with the WILDAPO group. The Hap1-Hap2 indicated the standardized haplotype distance of two haplotypes. The Hap2-WILDSEX1 indicated the standardized haplotype distance of Hap2 and the haplotypes from the WILDSEX1 group. (TIF) [file pgen.1010811.s024.tif]

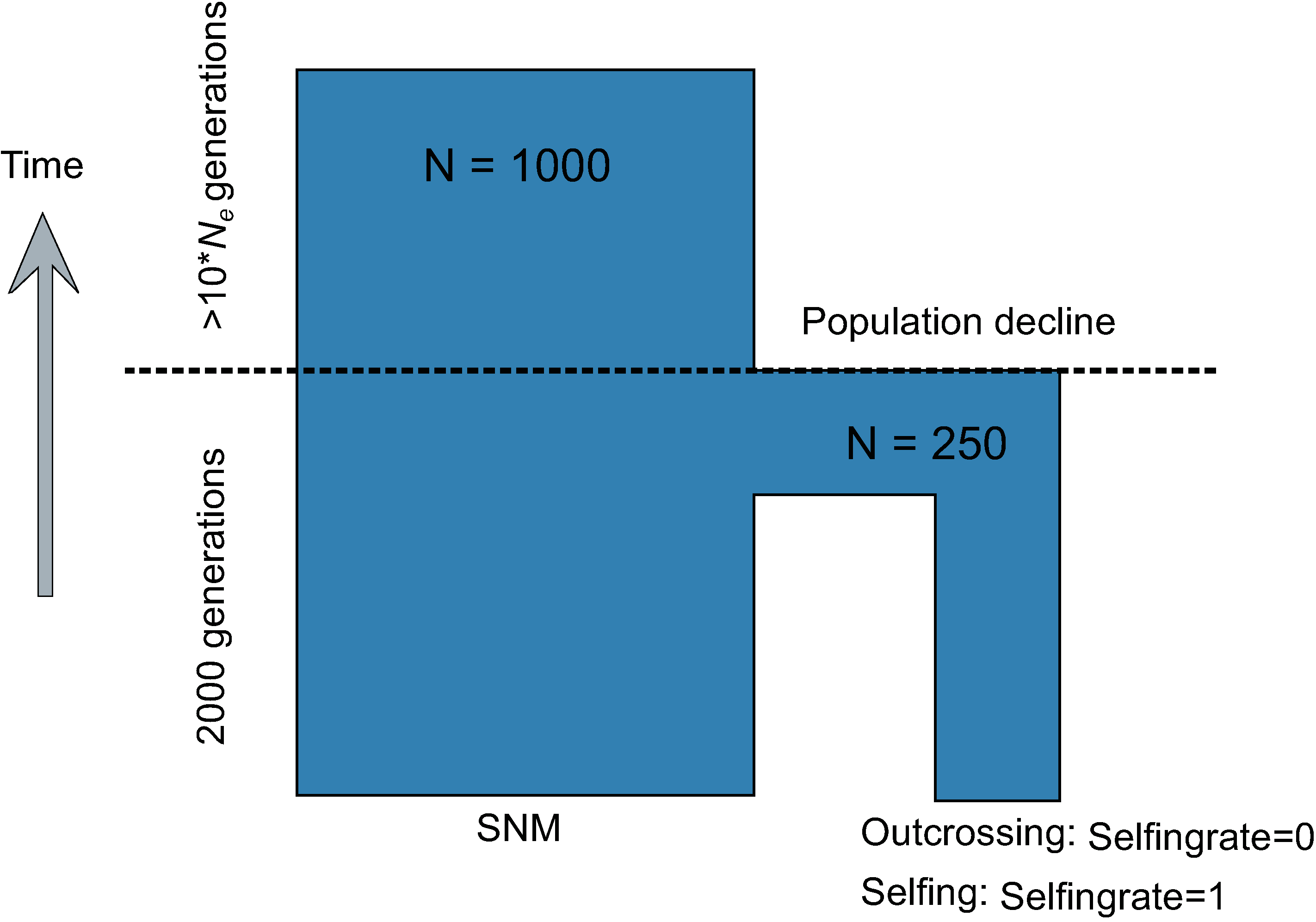

Supplement: S25 Fig — The demographic model used in forward simulation was inferred from the WILDSEX1 group using the SMC++program. Going forward in time, after a burn-in period of 10*N generations (100k generations), the ancestral sexual population splits into two subpopulations with 0.1*N population size, one capable of selfing (selfing rate = 1), one capable of outcrossing (selfing rate = 0) The dashed lines represent the time of demographic shift reproductive types. The parameters were simulated independently with 100 repeats. (TIF) [file pgen.1010811.s025.tif]

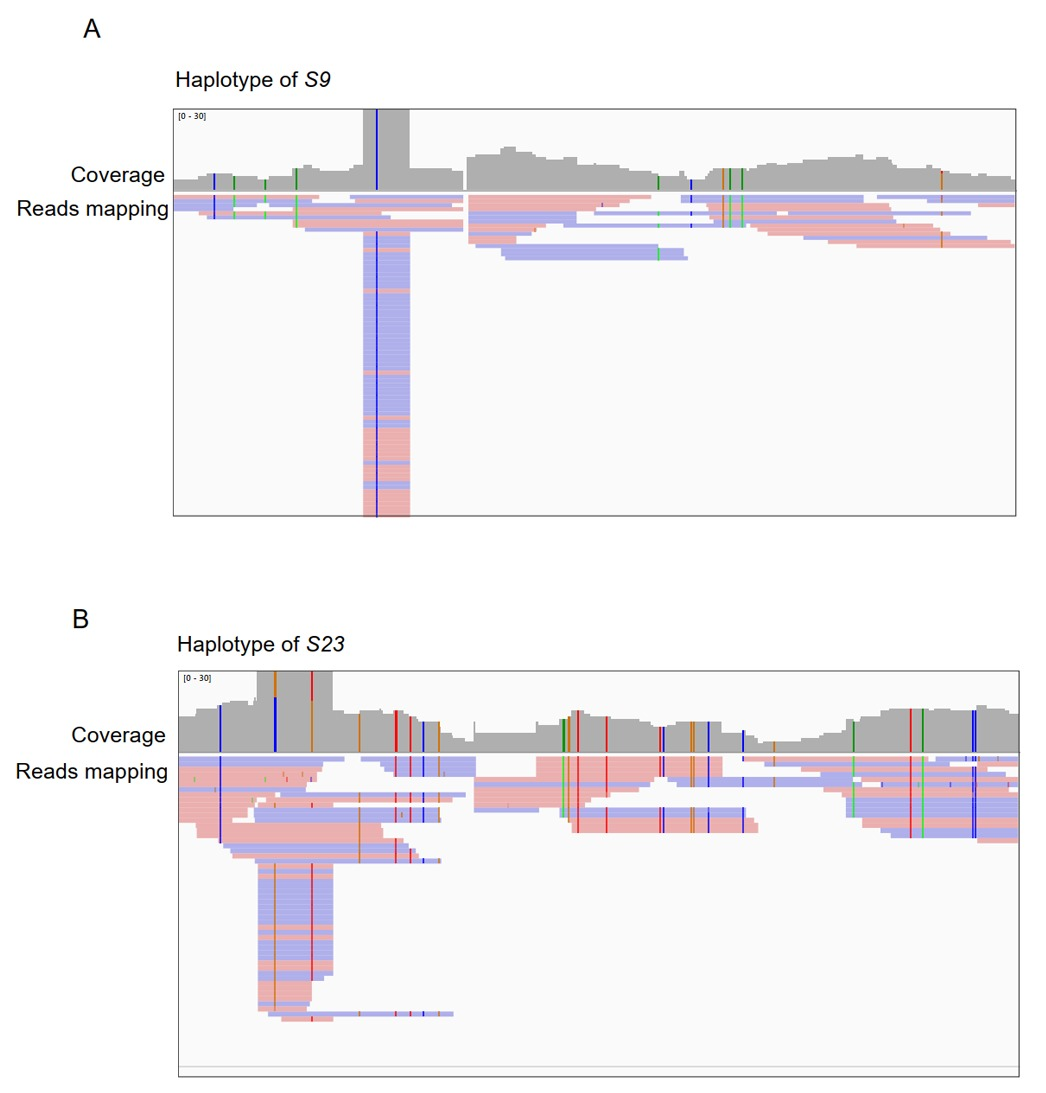

Supplement: S26 Fig — There were two S-alleles in the sexually reproducing sample ‘QLS’ from the WILDSEX2 group. (A) The IGV plot indicated the coverage of short-paired reads in the S9 sequences. The directions of reads were indicated by different colors. (B) The IGV plot indicated the coverage of short-paired reads in the S23 sequences. The directions of reads were indicated by different colors. (TIF) [file pgen.1010811.s026.tif]
